# Ultra-rapid somatic variant detection via real-time targeted amplicon sequencing

**DOI:** 10.1038/s42003-022-03657-6

**Published:** 2022-07-15

**Authors:** Jack Wadden, Brandon S. Newell, Joshua Bugbee, Vishal John, Amy K. Bruzek, Robert P. Dickson, Carl Koschmann, David Blaauw, Satish Narayanasamy, Reetuparna Das

**Affiliations:** 1 Department of Electrical Engineering and Computer Science, University of Michigan, Ann Arbor, MI 48109 USA; 2 Division of Computer Science and Engineering, Department of Electrical Engineering and Computer Science, University of Michigan, Ann Arbor, MI 48109 USA; 3 Division of Pediatric Hematology/Oncology, Department of Pediatrics, University of Michigan School of Medicine, Ann Arbor, MI 48109 USA; 4 Department of Neurosurgery, University of Michigan School of Medicine, Ann Arbor, MI 48109 USA; 5 Division of Pulmonary and Critical Care, Department of Internal Medicine, University of Michigan School of Medicine, Ann Arbor, MI 48109 USA

**Keywords:** Cancer screening, Assay systems, Sequencing, Computational biology and bioinformatics

## Abstract

Molecular markers are essential for cancer diagnosis, clinical trial enrollment, and some surgical decision making, motivating ultra-rapid, intraoperative variant detection. Sequencing-based detection is considered the gold standard approach, but typically takes hours to perform due to time-consuming DNA extraction, targeted amplification, and library preparation times. In this work, we present a proof-of-principle approach for sub-1 hour targeted variant detection using real-time DNA sequencers. By modifying existing protocols, optimizing for diagnostic time-to-result, we demonstrate confirmation of a hot-spot mutation from tumor tissue in ~52 minutes. To further reduce time, we explore rapid, targeted Loop-mediated Isothermal Amplification (LAMP) and design a bioinformatics tool—LAMPrey—to process sequenced LAMP product. LAMPrey’s concatemer aware alignment algorithm is designed to maximize recovery of diagnostically relevant information leading to a more rapid detection versus standard read alignment approaches. Using LAMPrey, we demonstrate confirmation of a hot-spot mutation (250x support) from tumor tissue in less than 30 minutes.

## Abstract (web summary)

A method for detection of somatic variants is presented for the intraoperative evaluation of tumor mutations in less than 30 min, using rapid DNA extraction, in silico optimized LAMP amplification, and a LAMP product analysis tool: LAMPrey.

## Introduction

Cancers are increasingly being diagnosed, characterized, and treated based on underlying genetic driver mutations^1^. Augmenting traditional histopathology with molecular diagnostics can not only improve diagnostic accuracy^2^, but also add prognostic value^3,4^ and inform disease management^5–7^. If diagnosed intraoperatively, during biopsy or resection, certain molecular markers can lead to a change in surgical management^6,8–10^, a combination of biopsy and resection procedures, and might even enable use of targeted, intraoperative therapies^11^. Molecular diagnostics can also guide rapid inclusion or exclusion from clinical trials that target certain mutation-specific pathways^5^. Intraoperative molecular diagnostics is an important tool in personalized medicine, motivating the development of rapid, and easy to perform molecular assays.

Despite their benefit, standard molecular diagnostics generally have long turn-around times^12,13^ (typically days to weeks) and usually cannot be performed within the intraoperative timeframe (which we define here to be <1 h). Prior attempts to fit molecular diagnostics within the intraoperative timeframe come with varying trade-offs (summarized in Supplementary Materials, Table S1). Intraoperative immunohistochemistry (IHC) can detect certain biomarkers that correlate to actionable mutations^14,15^, but is difficult to extend and apply to arbitrary mutations. Similarly, intraoperative Raman spectroscopy can quickly detect diseased tissue^16–18^, and is able to indirectly infer specific molecular markers such as IDH mutational status and 1p19q co-deletion^19^, but is currently not applicable to other mutations. Intraoperative fluorescent in-situ hybridization (FISH)^20,21^ and targeted quantitative polymerase chain reaction (qPCR) assays^8,22,23^ can provide rapid, and specific detection of targeted mutations or other cancer biomarkers. However, these techniques use allele specific fluorescent probes, and are limited to detection of a single allele change at a single locus^13^. All of these techniques are not easily extended to commonly mutated oncogenes where many possible allele changes (e.g. *KRAS p.G13, EGFR*) over a wide range of loci^24^ (e.g. *TP53)* would be clinically relevant^12,13^.

In contrast, targeted amplicon sequencing^12^ can identify any potential point mutation, small structural variants, copy number variations, and provide variant/mutant allele fraction (VAF), which has increasing clinical relevance for prognostics, disease tracking, and management^25,26^. Sequenced amplicons can also be informatically inspected to identify spurious, off-target amplification or other assay malfunction, increasing confidence in positive, negative, and indeterminant results. Yet even the fastest next generation sequencing-based (NGS) tissue-to-diagnostic protocols are currently much too slow to be used during surgery^12,13,27^. Furthermore, NGS sequencing is cost-optimized for sequencing of batched patient samples, and generally less accessible than other assay types, limiting the practical nature of intraoperative sequencing^12,13^.

**Fig. 1:**
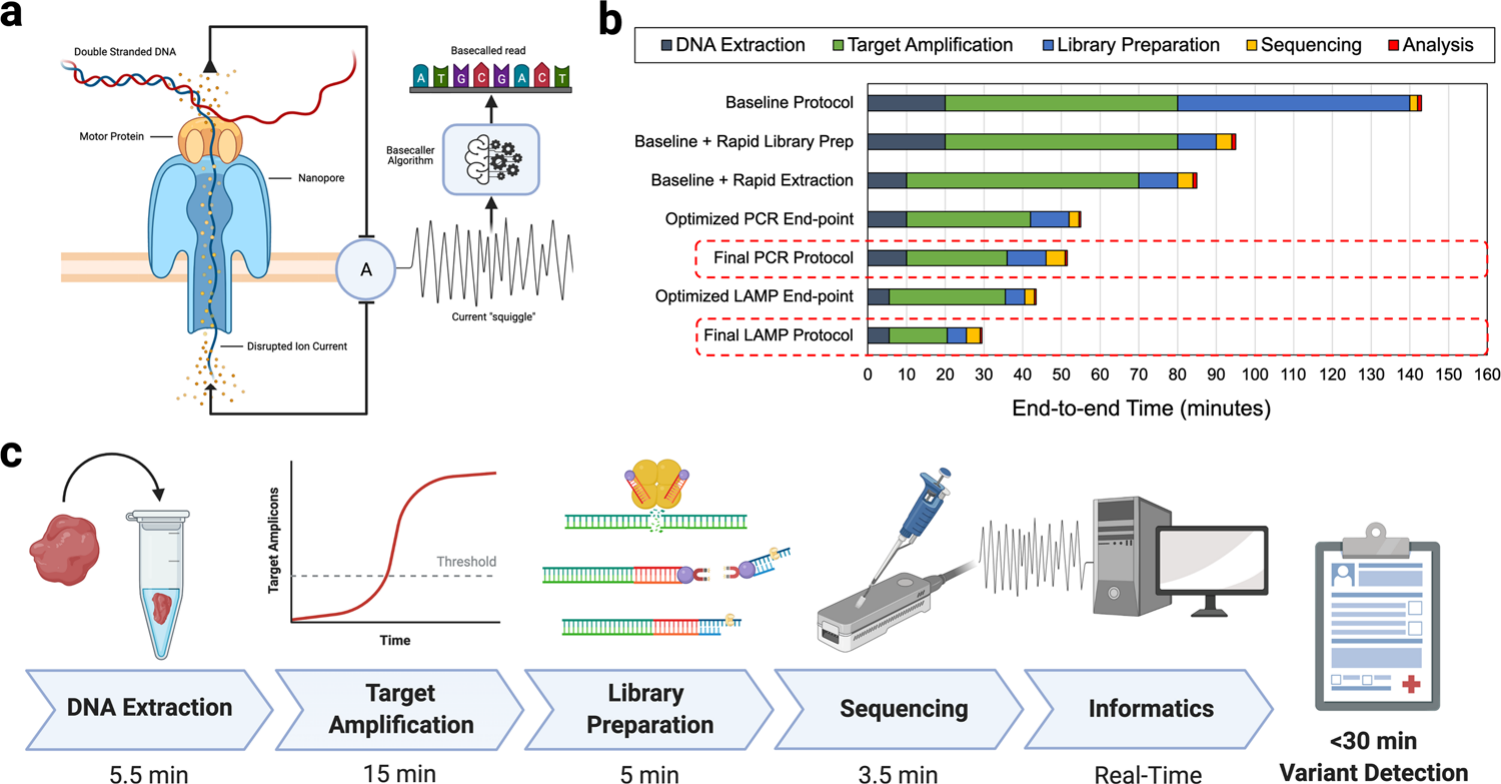
Overview of real-time Oxford Nanopore Sequencing, a time characterization of standard protocols and our optimization path, and an overview of our final, proof-of-principle diagnostic protocols. **a** Oxford Nanopore devices sequence DNA molecules by feeding a single strand through a pore embedded in a membrane. Disturbances in electrical current across the membrane correspond to individual base pairs and can be reconstructed (basecalled) to form the DNA sequence. ONT sequences provide streaming sequencing output in real-time enabling real-time bioinformatic analysis and diagnostics. **b** Characterization of time spent in various stages of ONT targeted sequencing pipelines. The baseline protocol is based off of standard suggested protocols for amplicon sequencing, and PCR cycling parameters used in prior work^28^. Coupled with easy-to-perform rapid extraction and library preparation protocols, we demonstrate that sequencing-based diagnostics can be performed within the intraoperative timeframe. **c** Optimized DNA extraction, LAMP amplification, and optimized library preparation can provide intraoperative sequencing-based diagnostic within 30 min.

Oxford Nanopore Technologies (ONT) has developed a portable, low-cost, small-form-factor sequencing device called the MinION that works by feeding DNA strands through nanopores embedded in a membrane^12,27^. The DNA disrupts ionic current flow across the membrane creating a signal that can be fed to a basecaller algorithm to recover the specific DNA sequence (Fig. 1a). ONT sequencers provide a rapid library preparation protocol, and streaming, real-time access to sequenced DNA for immediate analysis, which makes it a promising candidate approach for intraoperative sequencing^10,12^. Prior work using ONT sequencers performed targeted amplicon sequencing from pre-extracted DNA within ~2.5 h^28^. Newer work has explored untargeted, methylation-based classification of brain tumors within ~1.5–2.6 h^10^. However, a targeted, sequencing-based diagnostic within a more workable sub-1-hour intraoperative time-frame has yet to be demonstrated.

This work demonstrates a proof-of-principle approach for the development of ultra-rapid (<1 h) targeted sequencing-based molecular diagnostics. Time characterization of protocols from prior work and standard ONT sequencing pipelines identified two major time bottlenecks: library preparation and target amplification via PCR (Fig. 1b). To reduce both library preparation time and PCR time, we investigate the suitability of ONT’s rapid 10-min, fragmentation-based library preparation when applied to short, amplicons (<300 bp) and show that amplicons as small as 187 bp are detectable.

To further reduce overall time, we characterized assay amplification time and its impact on the amount of diagnostically useful product in the downstream sequencing library. Rather than rely on a fixed, end-point amplification protocol, we empirically identify an amount of target amplification (e.g. PCR cycles or time) that is estimated to lead to a time optimal diagnostic result for a particular assay. We then use this amplification threshold to guide a final protocol design. Using this methodology, we designed a proof-of-principle PCR-based protocol that was able to confirm the presence of a hotspot mutation from tumor tissue in ~52 min (Fig. 1b).

While rapid, amplification still accounts for over 50% of diagnostic time in our proof-of-principle PCR-based assay. To further reduce target amplification time, we investigate Loop-Mediated Isothermal Amplification^29^ (LAMP) as an alternative to PCR. LAMP uses isothermal strand-displacing polymerases, and intentional hairpin-forming primers to rapidly generate and extend concatemeric amplicons. LAMP offers much more rapid target amplification than traditional PCR (~30 min versus ~1 h), making it an ideal candidate for rapid molecular diagnostics^30–34^. However, LAMP assays are known to generate false positive amplification due to spurious mispriming events and primer self amplification^31,32,35^, thus, sequencing of LAMP product is recommended to inspect and identify proper amplification^31^. While some tools and protocols exist for sequencing and analysis of LAMP concatemers for COVID-19 diagnostics^31,36^, currently, there are no open-source bioinformatics tools designed to process complex LAMP concatemers (spurious or not) leading to difficulty in analyzing assay behavior, as well as missed diagnostic information, which results in a slower intraoperative diagnostic. We address these issues by designing an open-source LAMP concatemer analysis tool: LAMPrey (https://www.github.com/jackwadden/lamprey). LAMPrey diagnoses and classifies each input read according to expected design sequences and order generated by a properly behaved assay. Thus, LAMPrey allows for easy diagnosis of irregularities and inefficiencies in LAMP assays, including identification of suspected spurious amplification. Most importantly, LAMPrey can better identify diagnostically relevant target information when compared to the standard, suggested informatics pipeline for ONT-based reads. LAMPrey can also recover diagnostically relevant information lost to error-prone basecalling by aligning and comparing redundant sections of concatemers.

By time-optimizing DNA extraction and rapid library preparation protocols and using rapid LAMP amplification and the LAMPrey tool, we demonstrate a LAMP-based assay that can achieve 250x target locus support (mutant + wildtype calls) and successful somatic variant call from patient tumor tissue in less than 30 min (Fig. 1c). This timeframe is comparable or better than the fastest targeted molecular diagnostics, while also offering the benefits of a sequencing-based approach. These experiments demonstrate proof-of-principle that sequencing-based molecular diagnostics can be performed well within the intraoperative timeframe and have potential clinical utility.

## Results

### Rapid library preparation optimization

ONT’s recommended ligation-based library preparation (ONT #SQK-LSK109/110) takes between 40-60 min to perform and is assumed to be unsuitable for an intraoperative diagnostic^28,37^. Oxford Nanopore also offers a rapid library preparation chemistry^38^ (ONT #SQK-RAD004) that is advertised as a 2-step, 10-minute protocol. In the first step, DNA is simultaneously fragmented and tagged with click chemistry (tagmentation) via a transposome complex (5 min). In the second step, tagmented, double-stranded DNA is mixed with click-chemistry prepared ONT sequencing adapters and allowed to incubate for adapter attachment (5 min). This protocol is generally discouraged for use with short amplicons because of worry over the efficiency of the tagmentation reaction^39,40^. However, short amplicons are desirable for rapid targeted diagnostics because they generally allow for more rapid amplification.

To evaluate feasibility and trade-offs of using fragmentation-based library preparation with short amplicons, we designed, amplified, and sequenced various lengths of tailed PCR amplicons (187 bp, 260 bp, 609 bp, 910 bp) using the rapid protocol. Primers were designed to cover the clinically relevant *HIST1H3B K27M* hotspot mutation (listed in Supplementary Materials: Supplementary Note 1, Table S2, and Table S3). We chose to target this mutation because it is present in a large number of pediatric brain tumor samples available to us via a biorepository (see Methods).

We were successfully able to sequence and align all amplicons using standard bioinformatics pipelines (see Methods section). However, various inefficiencies were noted (described in detail in Supplementary Materials: Supplementary Note 2 and Figure S1). Our 260 bp assay had the best balance of amplification efficiency, and fragmentability, without over-fragmenting during library preparation. While longer amplicons are easier to fragment, they can fragment in multiple locations leading to a higher proportion of reads in the library that do not cover the locus of interest leading to a longer time-to-result. This assay was chosen for final cycle parameter optimization (Supplementary Material: Supplementary Note 3, Figure S2) and end-to-end diagnostic evaluation.

To reduce total library preparation time, we explored reducing the suggested rapid adapter incubation time and its impacts on relative sequencing performance (Supplementary Material: Supplementary Note 4, Figure S3). For four different incubation times (2–5 min) we identified no clear trend in relative sequencing performance within the first 10 min of sequencing. This indicates that a 2-minute incubation is at least sufficient to generate a sequencing library of acceptable quality for the ultra-rapid use-case, and further reduces the library preparation time by 3 min. This shortened protocol was used for the final LAMP-based demonstrations.

### Rapid DNA extraction evaluation

We chose to evaluate a rapid, 8-min DNA extraction kit—Lucigen QuickExtract^41^ (LQE)—which employs a one-pot protocol and can extract DNA from tissue that is immediately suitable for input to PCR amplification if diluted or directly as input for LAMP. The LQE protocol involves a 6-min extraction incubation and a follow-up 2-minute enzyme de-activation step. With physical overheads such as intermittent vortexing, pipetting, and handling, the protocol was easy to perform and took around 10 min in practice.

We then performed serial dilutions of the same LQE product to measure PCR amplification efficiency, and confirmed specificity and efficiency via gel electrophoresis (Supplementary Material: Supplementary Note 5, Figure S4). We also verified that this PCR product could be used as input to library preparation without an intermediary purification step (Supplementary Material Figure S5) and without unacceptable degradation of the flow cell membrane.

Further time reduction was achieved for LAMP amplification by shortening the DNA extraction incubation time from 6 min to 1 min and replacing the intermittent vortexing with a single initial 30 s vortex in a smaller volume of LQE (100ul) to quickly disrupt tumor tissue (Supplementary Material: Supplementary Note 6, Figure S6). LAMP amplification is both resilient to traditional PCR inhibitors and highly sensitive allowing us to amplify from small amounts of DNA. This shortened protocol was used for the final LAMP-based demonstrations.

### Timed PCR-based protocol demonstration

**Fig. 2:**
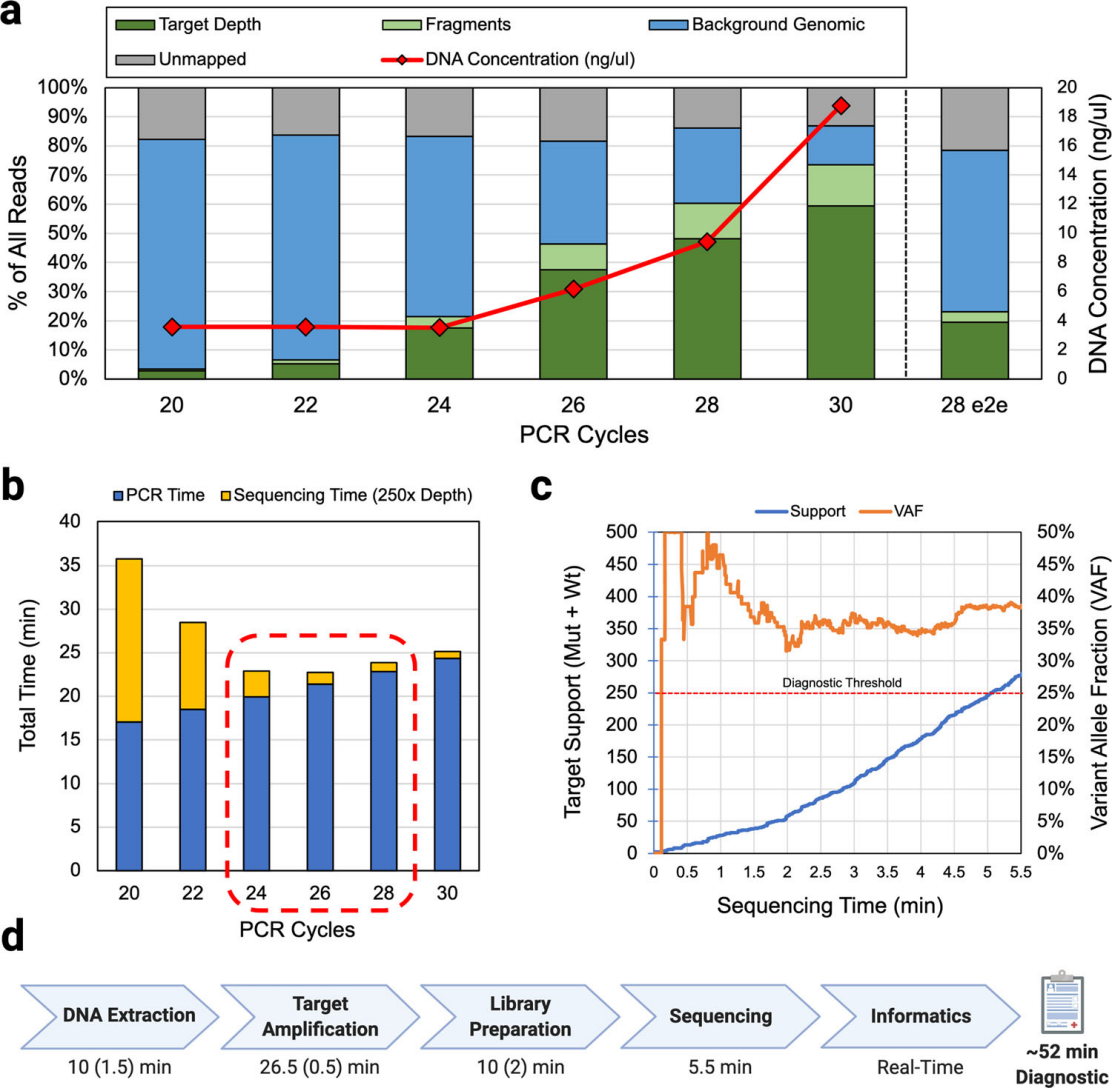
PCR assay performance characterization, optimal amplification time estimation, and end-to-end run results. **a** Sequencing results and DNA concentration from various cycle counts of PCR product and the final end-to-end demonstration (28 e2e). As DNA concentration increases, useful target fraction increases. **b** Amplification and sequencing time estimates corresponding to target fraction recovered from PCR cycle sweep suggest 24-28 cycles as the range of amplification times that result in the fastest end-to-end diagnostic result. Overprovisioning PCR cycles offers less of a time-penalty than under provisioning due to the exponential nature of PCR amplification and linear nature of ONT DNA sequencing. **c** In an end-to-end demonstration of the final protocol, using 28 cycles of PCR, we were able to reach 250x variant support within 5.5 min of sequencing time. **d** Approximate times for each diagnostic step with approximate manual overhead in parenthesis. Informatics was performed after the fact and verified to be performable in real-time with negligible overheads resulting in a ~52-min diagnostic.

A final amplification protocol was chosen by first characterizing the useful diagnostic fraction of reads generated over time by the optimized assay, estimating expected ONT MinION sequencer performance using an in-silico model, and finally choosing the amount of targeted amplification that results in the lowest predicted diagnostic time. Sequencing performance depends on a variety of factors including the number of pores available to sequence given a MinION flow-cell, the speed at which each pore is able to capture and sequence reads, and the required amount of read support to call a variant. The model used to estimate sequencing performance is discussed in Supplementary Materials (Supplementary Note 7, Figure S7, Table S4). PCR amplification efficiency was determined by performing our optimized PCR protocol for varying numbers of PCR cycles. We then measured both the resulting DNA concentration via Qubit fluorometer and sequenced each product (see Methods). Resulting reads were aligned to the human reference and classified according to alignment (see Methods). As expected, over time, both the total mass, as well as the proportion of target PCR product relative to background genomic reads (i.e. the useful diagnostic fraction) grow (Fig. 2a).

Assuming a variant call support (mutant + wildtype calls) requirement of 250x, our model estimated between 24 and 28 cycles as the amount of amplification that would result in the fastest end-to-end diagnostic result (Fig. 2b). 250x variant support was chosen as a semi-arbitrary threshold to allow for highly confident calls from common heterozygous somatic mutant allele fractions found from patient tumor samples (>10%). Given the current MinION sequencer error rate, 250x should provide between 99%-99.9% sensitivity and specificity assuming allele fractions from tissue samples are >10%. We discuss further motivation for picking 250x as a conservative diagnostic target and statistical methods in Supplementary Materials (Supplementary Note 8, Figure S8).

We then performed a timed run of this protocol starting with acquisition of ~20 mg of room temperature tumor tissue, performing rapid DNA extraction, 28 cycles of PCR amplification, ONT’s rapid library preparation, real-time sequencing (see Methods Section), and analysis to 250x target support (mutant + wildtype calls over the target). Because additional sequencing time penalty due to under-amplification is more severe than the time cost of extra PCR cycles, we chose to evaluate 28 cycles to account for unexpected inefficiencies and safeguard a result that fits within the intraoperative timeframe.

Target support over time and measured variant allele fraction are shown in Fig. 2c. Our protocol (see Methods) was able to achieve 250x support and successfully call a known *HIST1H3B K27M* variant from tumor tissue in ~52 min. Data analysis was performed retroactively but verified to be performable in near real-time. The approximate breakdown of time taken in each protocol step, including human overhead, is shown in Fig. 2d (see Methods; Supplementary Materials: Supplementary Note 9, Figure S9).

### LAMP assay development and efficiency evaluation

**Fig. 3:**
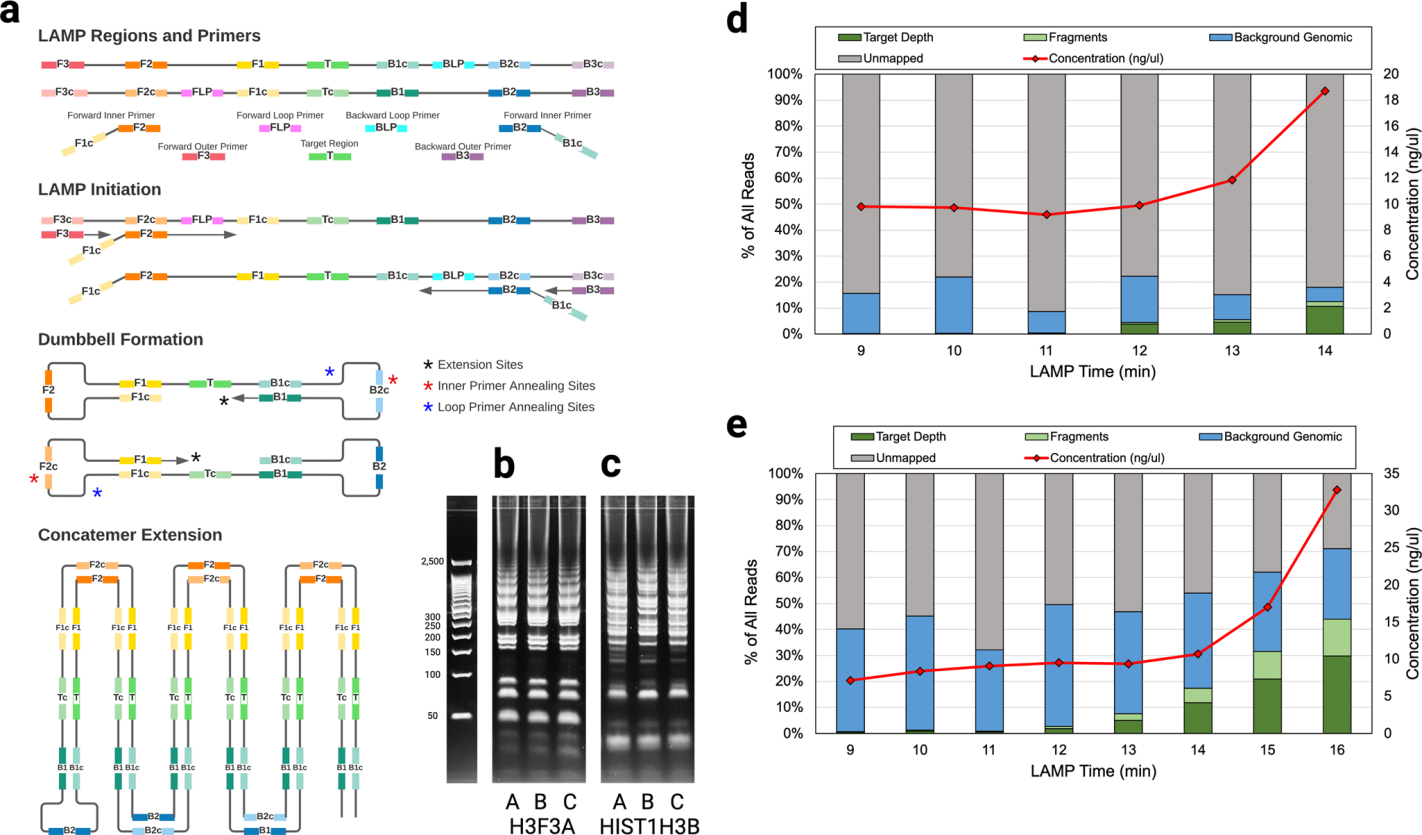
LAMP overview, and sequencing results from LAMP amplification timepoints. **a** LAMP amplifies a target region of DNA and forms concatemers by leveraging a strand-displacing polymerase and primers that form intentional hairpin loops. Multiple different concatemer types can be formed and extended. **b**, **c** Gel-electrophoresis of six runs of LAMP product reveals many different types of product and potential primer dimers and primer sequences. **b** Multiple runs on various input DNA samples shows the *H3F3A* assay forms clean bands, with concatemer types grouped according to integer multiples of the concatemer. The *HIST1H3B* product (**c**) displays similar patterns but seems contaminated by other spurious product and less organized. **d** DNA concentration measurements indicated large amounts of amplification of the *HIST1H3B* assay at 14 min, but sequencing confirmed a large proportion of that product was not able to be mapped to the human genome, suggesting spurious amplification or other assay malfunction along with a small amount of proper amplification. **e** The *H3F3A* assay produced a larger proportion of target product indicating better assay behavior.

Loop-mediated Isothermal Amplification^29^ (LAMP) offers more rapid target amplification than PCR and was chosen as a candidate to further reduce target amplification time. LAMP operates by carefully choosing a template region, design sequences (F3,F2,F1,B1,B2,B3), and primer sequences such that product intentionally forms self-hybridizing loops that result in the generation of concatemers (Fig. 3a). LAMP amplification has been used in a variety of use-cases where rapid time-to-result is important including pathogen detection^31,35^ and targeted-allele genotyping^34,42^. However, the complex machinery of LAMP can easily malfunction and lead to spurious amplification. By sequencing and properly analyzing LAMP product, we can diagnose issues with LAMP assays, and create both fast, and reliable assays.

To evaluate the feasibility of using LAMP as a replacement for PCR-based sequencing assays, we designed LAMP primer sets encompassing *H3F3A K27M* and *HIST1H3B K27M* hotspot mutations (see Methods) and evaluated each for amplification rate and specificity. These mutation targets were chosen because they are present in a large number of pediatric brain tumor samples available to us via a biorepository (see Methods section). LAMP primer sets were designed such that the target hotspot mutation was between the F2 and B2 regions (B1/B2 for the *H3F3A K27M* assay and F1/B1 for the *HIST1H3B K27M* assay) and not covered by the inner, or loop primers. Proper LAMP concatemer formation should form clear groups of bands corresponding to integer multiple concatemer amplicons. While the *H3F3A* assay formed clear groups, indicating proper amplification (Fig. 3b; Supplementary Figure S10) the *HIST1H3B* assay did not, indicating some spurious amplification or other partial assay malfunction (Fig. 3c; Supplementary Figure S10). Approximate amplification rate was measured by starting multiple LAMP reactions at the same time and removing and quenching reactions in ice at 1-minute intervals (see Methods). We then sequenced LAMP time interval product (see Methods) to investigate and measure LAMP product specificity. Both assays induced amplification—increasing DNA concentration over time—however bioinformatic analysis using Minimap2^43^ revealed that much of the *HIST1H3B* product generated was unmappable (Fig. 3d). In contrast, the *H3F3A* assay reliably amplified the target region and produced a more reasonable target fraction within 15 min (Fig. 3e). Existing standard read aligners like Minimap2 are not designed to consider concatemeric product, and the results provide little insight as to why the *HIST1H3B* assay malfunctioned. To the best of our knowledge, no prior work has developed tools for the analysis of LAMP concatemer product (other than ONT’s Guppy tool which is closed source and designed to process assays targeting COVID-19^31^).

### LAMPrey: a tool for LAMP product analysis and diagnosis

To investigate whether unmapped sequences were a failure of the LAMP assay, incompatibility between the LAMP product and ONT technology, or the applied bioinformatics toolchain, we designed a LAMP concatemer-aware alignment algorithm—LAMPrey. LAMPrey is designed to 1) diagnose the source of unmapped read, 2) to recover useful target information that standard long-read informatics pipelines miss, and 3) to leverage redundant information in concatemers to correct basecalling errors.

**Fig. 4:**
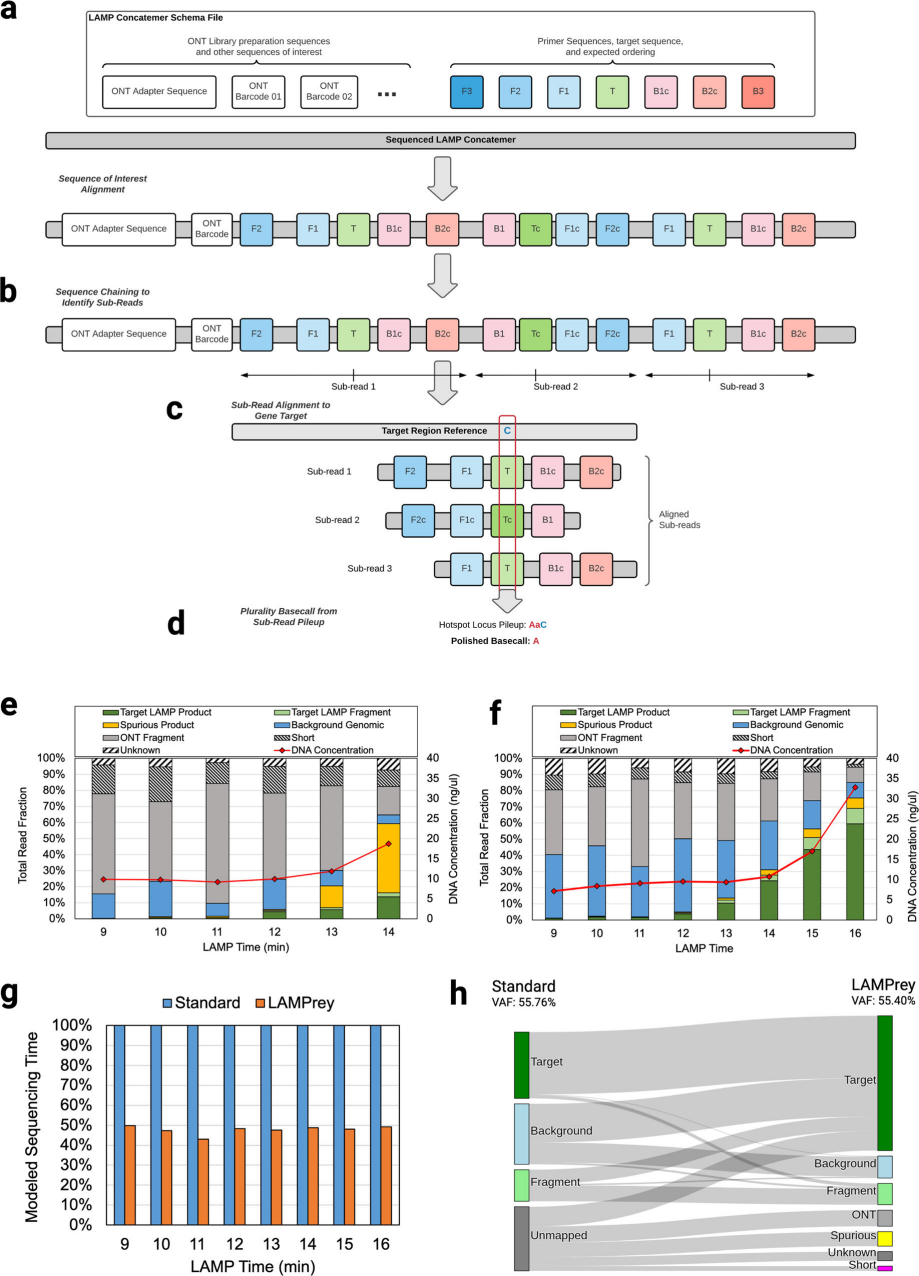
LAMPrey algorithm overview, classification results, and performance improvement relative to standard bioinformatics pipelines. **a** LAMPrey first marks suspected target regions and other sequences of interest in the candidate read. **b** Given a target instance, expected primer sequences are identified to the 5’ and 3’ ends of the target to form suspected concatemer sub-reads. **c** sub-reads are aligned to the gene target reference and (**d**) a consensus (plurality) agreement is applied to define a call over the locus of interest. LAMPrey is able to (**e**) help diagnose spurious LAMP amplification and recover diagnostically useful information from an otherwise malfunctioning assay or (**f**) confirm a properly functioning assay. **g** LAMPrey is able to recover more diagnostically relevant reads than standard bioinformatics pipelines, leading to a reduction in sequencing time required to reach desired target variant call support. **h** Classifications of all reads from the 16-minute *H3F3A* time-point using our standard pipeline (Minimap2) and LAMPrey. LAMPrey is able to recover a large amount of missed diagnostic information (i-iii) from reads missed by our standard pipeline. LAMPrey’s VAF (55.40%) is statistically identical to our standard approach (55.76%) indicating recovered information is not from an erroneous source.

LAMPrey begins by separately aligning the target region of interest, and all LAMP design sequences (F3, F2, F1, B1, B2, B3) to the read. All sequences that align to the read above a certain identity threshold (e.g. 70%) are marked as hits (Fig. 4a). This step is analogous to seeding in common genomic read mappers. If a target sequence is identified, LAMPrey looks to the left and right of the target for expected order of design sequences according to proper LAMP amplification (analogous to seed chaining). Correct sequences are incorporated into a sub-read candidate for each identified target (Fig. 4b). Once one or more sub-reads are identified, they are extracted and individually aligned to the region of interest using Minimap2^43^ (Fig. 4c). If a subread aligns, it is assumed to be properly formed and can be used for variant calling. If more than one sub-read aligns to the target, a pileup is generated and considered as a group for variant calling (Fig. 4d). A basecall is chosen via plurality of the calls in the pileup over the target. If there is a tie, a final call is randomly chosen between the tied plurality choices. This methodology (especially the sub-read identification and pileup for error correction) is similar to prior work leveraging rolling circle amplification (RCA) to generate concatemers and custom informatics to polish errors into a final consensus sequence^44–46^. These tools were considered for use but abandoned due to the inability to easily adapt each algorithm for use with LAMP concatemers.

After processing, LAMPrey classifies each read according to various heuristics. If an alignable target sequence is identified within the read, it is classified as *Target*. If the consensus read aligns to the assay target region, but does not contain the target, it is classified as *Fragment*. If no target sequence is identified, but many design sequence seeds (e.g. F2, F1) are identified, we assume the LAMP process malfunctioned generating spurious product, and the read is classified as *Spurious*. If few or no potential primer sequences are identified in the read, and the read successfully maps to the human genome, it is classified as *Background* genomic DNA. Lamprey also identifies reads that primarily contain ONT-related sequences such as adapters and barcodes (classified as *ONT*) and short fragments (<60 bp, classified as *Short*). The remaining reads are classified as *Unknown*. A more detailed description of the LAMPrey algorithm is provided in Supplementary Materials (Supplementary Note 10).

**Fig. 5:**
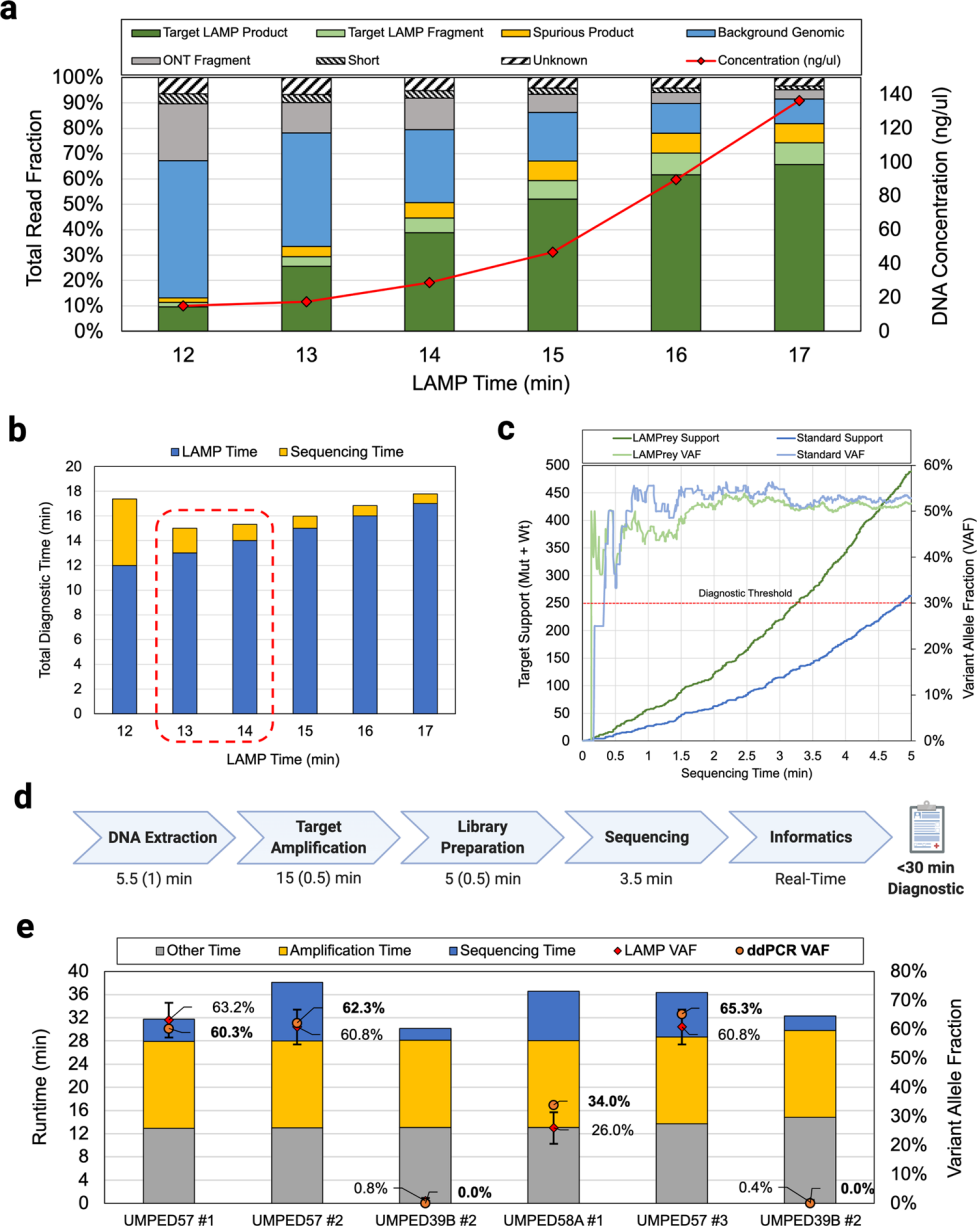
LAMPrey ultra-rapid sequencing assay results. **a** LAMPrey classification results from various amplification times and the final end-to-end demonstration (14 e2e) using DNA extracted from fresh tissue and the shortened LQE protocol. **b** Total sequencing time estimated from amplicon fraction at various LAMP time points. Our model predicted that 13–14 min of LAMP would lead to the fastest end-to-end diagnostic. We chose to evaluate 14 min to guard against under-amplification or other sequencer performance model misprediction error. **c** Diagnostic results of the 14-min end-to-end LAMP assay over time. A standard bioinformatics pipeline failed to leverage ~48% of target reads. LAMPrey can recover this information and leads to a diagnosis ~96 s sooner. **d** Approximate times for each diagnostic step with approximate manual overhead in parenthesis. Library preparation is notably faster than the 3-step PCR evaluation due to reduced adapter incubation time and ice quenches. Amplification time is lower due to our 14-minute LAMP protocol vs ~26-minute (28 cycle) PCR amplification protocol. **e** Results from barcoded protocol runs after prediction model refinement showing results over multiple patients (*n* = 3), samples (*n* = 6), and flow cells (*n* = 3). Replicates were performed with barcoded library preparation kit and without ice-plunges. An updated sequencer performance model estimated 15-minutes as the optimal amount of amplification. VAFs reported by ddPCR using the same DNA from each ultra-rapid run closely match those reported by LAMPrey (error bars computed using two-sided proportion confidence interval CL = 95%).

We used LAMPrey to characterize sequenced LAMP product from each time point from the amplification time sweep runs of both *HIST1H3B* and *H3F3A* primer sets. For the *HIST1H3B* assay, LAMPrey was able to identify large amounts of spurious amplification (Fig. 5e) which indicates a dysfunctional LAMP assay. In contrast, the *H3F3A* assay generates a consistently healthy proportion of on-target LAMP product and was chosen for our final end-to-end demonstration (Fig. 4f). However, it is worth noting that by sequencing LAMP product and leveraging the LAMPrey tool, the *HIST1H3B* assay is still usable within the intraoperative timeframe, albeit with a time penalty due to wasted time sequencing spurious product.

When applied to the time-sweep sequencing results, LAMPrey was able to recover more than twice as many diagnostically relevant reads resulting in ~2x improvement in modeled sequencing time-to-result (Fig. 4g). To identify the source of benefit, we manually analyzed reads with diagnostically relevant information that Minimap2 missed, but LAMPrey was able to recover (Fig. 4h, i-iii). Most missed diagnostic information was due to imbalanced concatemers where a longer, higher quality concatemer section that did not cover the target region of interest was chosen as the primary mapping over a shorter concatemer section that included the diagnostically relevant hotspot. These sources of missed diagnostic information are discussed in detail in Supplementary Materials (Supplementary Note 11).

### Timed LAMP-based protocol demonstration

Similar to our PCR-based proof-of-principle, a final LAMP-based amplification protocol was chosen by first characterizing the useful diagnostic fraction of reads generated over time. LAMP amplification efficiency was determined by performing our LAMP protocol for varying amounts of time and sequencing the results (Fig. 5a). Resulting reads were classified using the LAMPrey tool to estimate the useful diagnostic fraction over time. Assuming a variant call support requirement of 250x, our sequencing time model estimated between 13-14 min as the amount of amplification that would result in the fastest end-to-end diagnostic result (Fig. 5b). Because additional sequencing time penalty due to under-amplification is more severe than the time cost of extra LAMP amplification time, we chose to evaluate a relatively conservative LAMP amplification time of 14 min. These libraries and the end-to-end sequencing demonstration were prepared using the 3-minute rapid DNA extraction protocol and 2-min rapid adapter incubation library preparation protocol previously identified as appropriate.

We then performed a timed, end-to-end run using ~20 mg of tumor tissue as input. Diagnostically useful read support over time and sample variant allele fraction is shown in Fig. 5c. The protocol (see Methods section) was able to achieve 250x coverage and successfully call a known *H3F3A K27M* variant from a pediatric tumor sample in ~29.5 min. Using the LAMPrey tool, we were able to recover ~40% more information than the standard approach leading to a savings of ~1.5 min of sequencing time (Fig. 5c). The approximate breakdown of times for each step, including human overhead, is shown in Fig. 5d. The detailed protocol is shown in Supplementary Material (Supplementary Note 12, Figure S11) and available online (see Methods Section). To the best of our knowledge, this is the first sub-30-minute sequencing-based variant call achieved to date and demonstrates the feasibility of using sequencing as a diagnostic technique within the intraoperative timeframe.

To evaluate assay accuracy and variance over tissue samples and flow cells, we repeated our LAMP-based protocol on tissue samples from different patients (*n* = 3), tissue aliquots (*n* = 6), and flow cells (*n* = 3). To mitigate inter-run contamination when re-using flow cells, we used unique barcoded sequencing adapters (#ONT SQK-RBK004) and washed flow cells with a nuclease flush (#ONT EXP-WSH004) between runs. The sequencing performance estimation model was modified with new parameters to account for efficiency losses due to incorrect barcode identification and also low pore participation common within the first few minutes of sequencing. These parameters were monitored between runs and adjusted in an attempt to identify reasonable values representative of average behavior. Ice-plunges were replaced with thermocycler programming to reduce probability of human error, at the cost of ~30 additional seconds. The LAMPrey tool was also modified to improve performance and basecall and analyze sequencer output in real time. Guppy 5.11 was also used. These changes resulted in slightly different target fractions that were used in the updated sequencing time prediction model. Runtimes and results for each run are shown in Fig. 5e.

Three technical replicates (runs 1, 2, 5; UMPED57) were performed on separate aliquots of tissue from the same *H3F3A K27M* positive tumor used in the initial proof-of-principle run. Biological replicates were performed on one other *H3F3A K27M* positive patient sample (run 4; UMPED58A) and one *H3F3A K27M* negative patient sample (runs 3,6; UMPED39B). For known negative samples, LAMPrey reported slight positive allele fractions (0.4–0.8%), which roughly corresponds to the previously characterized false positive error rate (~1%) of the MinION sequencer for this allele at this locus^37^. This indicates that our approach is able to confidently identify negative samples. Resulting DNA was cleaned and analyzed using a droplet digital PCR (ddPCR) assay (see Methods Section). ddPCR VAFs closely matched LAMPrey VAFs in 5/6 runs (Fig. 5e; error-bar CI = 95%). An 8% difference was noted in run 3 indicating possible sampling or amplification bias introduced by LAMP or ddPCR amplification.

Flow cells were re-used once to investigate how inter-run flow-cell degradation and contamination would influence performance or reported VAF. Runs performed on re-used flow-cells (runs 2,4,6) tended to have higher runtimes due to fewer pores being available but were still able to produce results well within the intraoperative time-frame. Inter-run contamination was identified by a relatively high-proportion of barcoded reads from initial runs appearing in results after re-use. However, we suspect most contamination was able to be resolved informatically, indicated by the strong negative in run 6 after an initial positive in run 5. However, inter-run contamination could explain the lower-than-expected VAF reported by LAMPrey in run 4. In all cases, the assay was able to confidently diagnose disease within the intraoperative time-frame, with an average time-to-result of 34 m12 s.

## Discussion

To the best of our knowledge, this proof-of-principle work is the first demonstration of a repeatable, sequencing-based molecular diagnostic within a sub-1 h intraoperative timeframe. By co-optimizing DNA extraction, target amplification, library preparation, and informatics, we were able to achieve a sequencing-based variant call from real patient tissue within 30 min.

The sequencing performance model was reasonably successful at helping design ultra-rapid protocols within a set end-to-end time-frame, with total protocol prediction error ranging from ~1% (20 s) to ~11.6% (~4 m15 s). However, it was difficult in practice to accurately and precisely predict sequencing time. This was mostly due to variability in LAMP assay amplification rate and difficulty predicting ONT sequencing rate. LAMP amplification rate depends on factors such as the quality, amount, and composition of the tissue aliquot, as well as the fidelity of the pre-characterization of amplification rate (including basecaller accuracy). In practice, we chose relatively conservative amplification times for final evaluation that favored over-amplification in order to produce a more repeatable end-to-end runtime given our difficulty accurately predicting these factors. Future implementations could leverage real-time amplification measurement to identify when a desired amplification threshold has been reached in real-time. ONT sequencing rate was also difficult to predict in practice, and depends on multiple factors that are difficult to control or model such as pipetting error, proper library mixing with loading beads, and the even distribution of the library over the flow-cell during loading. Future work should attempt to identify variables that have a large impact on sequencing performance to allow for more accurate and precise predictions. Regardless of the difficulty in predicting sequencer performance, the model proved a useful tool for suggesting a range of protocols for final evaluation.

While this work focused on designing assays for *HIST1H3B K27M* and *H3F3A K27M* histone mutations—common in pediatric diffuse midline gliomas available to us via a biobank—this assay design and optimization approach could be applied to any nucleic acid amplification assay that targets other hotspot mutations or oncogenes. Each new assay will require an initial time-sweep characterization and target fraction determination, but can otherwise use the same DNA extraction and library preparation protocols developed in this work. Mutation hotspots such as *BRAFV600*, *IDH1R132*, *IDH2R172*, and *IDH2R140* are prime examples where identifying mutational status during surgery could inform more-aggressive resection (*BRAF mutant*) or less-aggressive resection (*IDH1/2 mutant*) of gliomas.

While our current realization is a singleplex assay and requires ~14–15 min of amplification time, higher throughput devices on ONT’s future product roadmap—with orders of magnitude more parallel pores—would allow for a more rapid time-to-result, and/or ultra-rapid multiplexed assays. Multiplexing could be accomplished within the same reactor, or in separate reactors with each product combined before library preparation. Given the different amplification rates and useful target fractions of various assays, our sequencer performance model would need to be modified to balance the sequencing rate of each target. With the use of a barcodes, suspected tumor tissue could be sequenced along with normal tissue providing a simultaneous negative control. Matched tumor/normal sequencing would also enable more accurate variant calling, and even copy number variation analysis. Multiple tissue samples from various locations in the tumor could be sequenced providing a spatial map of tumor genetics. In this manner, multiplexed ultra-rapid sequencing could quantitatively outline tumor margin and map potential molecular heterogeneity intraoperatively.

The protocols developed in this work are low-complexity, and all required equipment could easily fit onto a surgical cart. This would allow the assay to be practically performed within an operating room or adjoining pathological suite without the overhead of moving the sample to a dedicated diagnostic laboratory. We also expect these protocols to be automatable and performable using laboratory robots or microfluidic labs-on-a-chip (e.g. ONT’s Voltrax device). Automation would further reduce expertise required to perform the assay, reduce human error and inefficiencies, and reduce total required equipment footprint.

Due to the low-cost of the MinION sequencing device (~$1000), total equipment costs are low relative to NGS sequencing. However, per-run costs can be as high as ~$625 ($475/flow-cell + ~$150/reagents). Assuming re-use, per-run costs are estimated to be ~$387.5 ($237.5/flow-cell + ~$150/reagents). While not insignificant, we anticipate assay costs to be easily justified by savings associated with reduction in operating room time (from combining surgeries) and improvements to patient outcomes. We also anticipate that flow-cell costs will decrease significantly in the future as low-cost, single-use flow cells improve in reliability and capability.

By using the LAMPrey tool to polish LAMP concatemers with multiple alignable target regions, we were able to filter out some known or suspected incorrect basecalls. This self-polishing feature could be leveraged in the future to provide highly accurate variant calls at lower coverage, and for use-cases where variant allele fractions are near or lower than the current ONT sequencer error rate (e.g. liquid biopsy^46^).

The LAMPrey tool was also able to recover more information than minimap2 in our evaluation (~2x for the *H3F3A K27M* assay evaluated). As minimap2 was not designed to account for complex concatemeric product, it is not surprising that LAMPrey was able to outperform it. These results clearly motivate the use of tools and algorithms designed to process LAMP concatemers. We hope that this work will establish LAMPrey as an appropriate baseline for continued development of LAMP concatemer aware tools in the future.

## Methods

### PCR primer design and size evaluation

PCR primer sets were drawn from multiple sources and selected or designed to flank the *HIST1H3B K27M* hotspot mutation. The 260 bp primer set used in the ultra-rapid end-to-end demonstratiion was designed using Primer3 v4.1.0 and tailed at the 5’ ends with ONT handshake sequences (bolded) to enable potential interoperability with ONT’s four-primer library preparation protocol (Forward:5‘-**TTTCTGTTGGTGCTGATATTGC**GAATCAGCAACTCGGTCGAC- 3’, Reverse: 5’-**ACTTGCCTGTCGCTCTATCTTC**CAGGCAAGCTTTTCTGTGGT-3’). All primers were supplied by Integrated DNA technologies. PCR was performed using New England Biolabs (NEB) Q5 2x Master mix (NEB #M0492) and standard primer concentrations. 30 ng of NA12878 DNA (Coriell Institute) was used as template and 35 cycles of PCR was performed on a BioRad C1000 thermocycler with the following cycling parameters: Initial denaturation for 30 s @ 98 °C, 35 cycles of 10 s @ 98 °C, 15 s @ 58 °C-68 °C, 40 s @ 72 °C, and a final extension at 72 °C for 2 min. Library preparation was performed using Oxford Nanopore’s Rapid Barcoding Kit (ONT #SQK-RBK004) and sequenced using a MinION R9.4.1 flow cell. Fast5 signal files were basecalled using ONT’s GPU accelerated basecaller Guppy version 4.2.2. Reads were aligned using Minimap2 version 2.17 (-x map-ont) against the human reference genome (GRCh37/hg19). Alignments were filtered for primary alignments using samtools^47^ v1.7 (-F 0 × 900). Alignments were classified using to a custom script (see Code Availability). All primer sets are listed in Supplementary Materials Table S1.

### DNA extraction

DNA was extracted from samples taken from diffuse midline gliomas originally acquired at autopsy, flash frozen, and stored at −80 °C. Sub-aliquots were divided into sections between 20-30 mg and stored at −20 °C before experimentation. DNA extraction was performed after tissue was allowed to thaw to room temperature. For PCR experiments targeting the *HIST1H3B K27M* variant, we used Lucigen QuickExtract DNA extraction solution (Lucigen #QE0905T) and the standard protocol was followed. ~20–30 mg of tissue was placed in 500ul of Lucigen QuickExtract solution in a 2 ml Eppendorf tube and vortexed for 15 s. The solution was incubated for 6 min at 65 °C, vortexing briefly after 3 min and 6 min. The solution was finally incubated at 98 °C for 2 min before either being 1) immediately used for amplification or 2) stored at 4 °C for later amplification. For LAMP experiments targeting the *H3F3A K27M* variant, ~20–30 mg of tissue was placed in 200ul Lucigen QuickExtract solution in a 1.5 ml tube and processed as above.

The Lucigen QuickExtract Protocol was optimized by shrinking the incubation time to 1 min and increasing the initial vortex time to 30 s and eliminating intermittent vortexing steps. The optimized protocol also uses 100ul of LQE per ~20 mg aliquot of tissue and uses 0.2ul PCR tubes. This allows the user to use one thermocycler for extraction, amplification, and library preparation, obviating the use of multiple heat blocks. This optimized methodology was used in the final end-to-end LAMP experiment and all replicates.

### PCR cycle-sweep amplification and sequencing

PCR reactions were prepared according to the NEB Q5 2x master mix (NEB #M0492) protocol using a 1ul of LQE extracted DNA product as input (~20 ng DNA). PCR of varying cycles was run sequentially on a BioRad C1000 thermocycler with a 3 °C/s ramp rate with the following cycling parameters for 20, 22, 24, 26, 28, and 30 cycles: Initial denaturation for 30 s @ 98 °C, *N* cycles of 5 s @ 98 °C, 5 s @ 64 °C, 8 s @ 72 °C, and a final extension at 72 °C for 2 min. The sequencing library was prepared according to the ONT rapid barcoding kit protocol (ONT #SQK-RBK004) with separate barcodes assigned for each PCR cycle number and sequenced on a MinION R9.4.1 flow-cell. Resulting Fast5 signal files were basecalled using ONT’s GPU accelerated basecaller Guppy version 4.2.2. Reads were aligned using Minimap2 version 2.17 (-x map-ont) against the human reference genome (GRCh37/hg19). Alignments were filtered for primary alignments using samtools v1.7 (-F 0×900) and classified according to alignment position and pileup results using a custom script (see Code Availability). Post-amplification DNA concentration was measured via Qubit (Invitrogen dsDNA HS assay #Q33230).

### PCR-based ultra-rapid sequencing protocol

A full protocol is available at https://www.protocols.io/view/ultra-rapid-sequencing-pcr-bs7bnhin. Briefly, tumor tissue was allowed to thaw and come to room temperature. Using 20–30 mg of tumor tissue as input, DNA extraction was performed using Lucigen QuickExtract DNA extraction solution (Lucigen #QE0905T) and the standard protocol was followed, followed by a 30 s ice quench. 1 ul of extracted DNA was added to a tube with 24ul premixed PCR master mix, primers, and water (according to the standard 25ul Q5 2x Master Mix protocol) and placed in a thermocycler for amplification. During amplification, a sequencing run in the ONT MinKNOW software was started and the MinION flow cell was initiated (pore health check and initial mux scan) and primed. A sequencing run was started without a library and paused using the MinKNOW software. After the PCR protocol finished, 7.5ul of PCR product was immediately added to 2.5 ul of fragmentation mix and tagmentation was initiated in the same thermocycler according the ONT Rapid Library Preparation protocol (ONT #SQK-RAD004). Rapid sequencing adapter was added (1ul), and allowed to incubate for 5 min. With 1-min remaining, the flow cell was re-primed for library loading. The library was then immediately mixed and the run unpaused in MinKNOW. Informatics (basecalling, alignment, variant calling) was performed after the fact. Fast5 files were basecalled using Guppy version 4.2.2 and reads were aligned using Minimap2 version 2.17 (-x map-ont) against the human reference genome (GRCh37/hg19). Alignments were filtered for primary alignments using samtools v1.7 (-F 0 × 900) and classified according to alignment position and pileup results using a custom script (see Code Availability). Time to variant call was computed from FASTQ reads sorted by ONT read start time timestamps and extracted using a custom script (see Code Availability). For off-line analysis, we assume the start time recorded as a part of read metadata approximates data availability given that a vast majority of sequenced reads take <1 s to sequence, and the rapidity of basecalling/analysis. In reality there will be some cost for streaming analysis. Analysis of all data required to reach the 250x depth requirement took ~27.7 s on an available server housing an Intel Xeon W-2133 3.6 GHz CPU and an NVIDIA Quadro P2000 GPU. Thus, we expect streaming analysis to cost at most 30 s, and is more likely an insignificant additional time cost relative to the measured 52-minute protocol.

### LAMP primer design and time sweep

LAMP primer sets were designed using Premier Biosoft LAMP Designer v1.16 trial version and evaluated for efficiency and specificity using both gel electrophoresis and sequencing. The following primer sets were chosen for final evaluation with inner-primer poly-T linker sequences bolded: *HIST1H3B K27M*: F3- GATCGGTCTTGAAGTCTTGG, B3- ATAGTTGGTGGTCTGACTCTAT, FIP- AAAGCCTCACCGTTACCG**TTTT**CCGAATCAGCAACTCG, BIP- GAGCAGCCTTGGTAGCCAG**TTTT**GGCTCGTACTAAACAGACAG, FLP- GAGATCCGCCGCTACCAA, BLP- TTACCGCCGGTGGATTTC. *H3F3A K27M*: F3- GTTTGGTAGTTGCATATGGTG, B3- ATACCTGTAACGATGAGGTTTC, FIP- GCGGGCAGTCTGCTTTGTA**TTTT**ATGCTGGTAGGTAAGTAAGGA, BIP- CGACCGGTGGTAAAGCACC**TTTT**CACCCCTCCAGTAGAG, FLP- CGAGCCATGGTACAGAGAC, BLP- CAGGAAGCAACTGGCTACA.

LAMP amplification was performed (NEB #E1700) following the standard protocol but omitting final polymerase deactivation. Separate reactions were prepared using the *HIST1H3B* primer set and run simultaneously. Reactions were pulled and quenched at various time points to stop amplification. The sequencing library was prepared according to the ONT rapid barcoding kit protocol (ONT #SQK-RBK004) assigning separate barcodes to each time-point and sequenced on a MinION R9.4.1 flow-cell. Resulting Fast5 signal files were basecalled (Guppy v4.2.2) and reads were aligned to the human reference genome (GRCh37/hg19; Minimap2 v2.17 -x map-ont) against the human reference genome and filtered for primary alignments (samtools v1.7 -F 0×900). Post-amplification DNA concentration was measured via Qubit (Invitrogen dsDNA HS assay #Q33230).

### LAMP-based ultra-rapid sequencing protocol

A full detailed protocol is available at https://www.protocols.io/view/ultra-rapid-sequencing-lamp-btvmnn46. Briefly, prior to beginning, the MinION flow cell was initiated (pore health check and initial mux scan) and primed. A sequencing run was started without a library and paused using the MinKNOW software. LAMP, tagmentation, and sequencing mixes were prepared. The run timer was started and DNA extraction was performed. 1 ul of extracted DNA was sampled at room temperature and immediately amplified via LAMP in a thermocycler at 65 C for the amplification time suggested by the sequencer performance model. After amplification, LAMP product was cooled to room temperature (via ice plunge or thermocycler draw-down) and 1.9 ul of product was added to 2.5ul of ONT fragmentation mix (FRA) and 5.6 ul of nuclease free water. Tagmentation was initiated in the same thermocycler according to the ONT protocol (ONT #SQK-RAD004). After cooling to room temperature (via ice plunge or thermocycler draw-down), 1ul of ONT rapid sequencing adapter (RAP) was added and allowed to incubate for 2 min. During the final minute of incubation, the flow cell was re-primed. The DNA library was then mixed with the sequencing mix and loaded onto the flow-cell according to the standard protocol. The sequencing run was then re-started to initiate sequencing. For the initial proof-of-principle run, informatics (basecalling, alignment, variant calling) was performed after the fact but verified to be performable in real-time. Fast5 files were basecalled and fastq files processed as in the PCR-based approach or using the LAMPrey tool. To adjust model parameters, available/active pores and per channel sequencing rate was computed using the sequencing rate script (see Code Availability) for each run. For replicate runs, sequencing rates were updated after each run to reflect the best estimate of expected flow-cell performance, adjusting the model after each run. Time-to-variant call was computed from FASTQ reads sorted by ONT read start time timestamps and extracted using a custom script or via the LAMPrey tool (see Code Availability). For this off-line analysis, we assume the Guppy basecaller *start_time* approximates data availability and variant call given that a vast majority of sequenced reads take <1 s to sequence, and the rapidity of basecalling/analysis. For the LAMP replicate runs, LAMPrey was run in online mode, computing results in real time and using Guppy version 5.0.11.

### Droplet digital PCR (ddPCR) confirmation of variant allele fractions

After running the LAMP-based ultra-rapid protocol, the tube containing the tissue aliquot and 100ul of the extracted DNA solution was briefly centrifuged. 80-100ul of the QuickExtract solution was removed (being careful to avoid any tissue) and deposited into a 1.5 ml Eppendorf tube. The DNA from this solution was purified using Qiagen DNeasy Blood and Tissue kit (QIAGEN #69504) with a modified protocol. Briefly, 20ul of proteinase K mixed with the extracted DNA and vortexed on high. After incubation at room temperature for 1 min, 200ul of PBS was added to the mixture and mixed by vortexing. 200ul of 100% ethanol was added and the mixture was vortexed on high. The remainder of the Qiagen protocol was followed according to the manufacturer’s instructions for tissue-based extractions. ddPCR was performed using the following forward and reverse primers (Forward: CTCTGTACCATGGCTCGTA, Reverse: CATACAAGAGAGACTTTGTCCC, Wt Probe: 5’ - /56-FAM/ TC+GC+A+T+GA+GTGC /3IABkFq/ -3’, Mut Probe: 5’ - /56-FAM/ TC+GC+A+A+GA+GTGC /3IABkFq/ - 3’) and protocol outlined in Cantor et al.^48^. Briefly, triplicate reactions were prepared with purified DNA from each ultra-rapid LAMP replicate run. Droplets were generated and read using a BioRad QX200 system. Results were analyzed using QuantaSoft v1.0.596. VAFs were determined by isolating distinct populations of droplets and recording fractional abundance reported by the software. Positive *H3F3A K27M* VAFs were reported if at least three positive droplets were identified.

### Statistics and reproducibility

All error bars for ultra-rapid runs were calculated using the confidence interval for a population proportion for a given confidence level. Both time-to-result and allele fractions for technical and biological replicates were found to be easily reproducible given that proper attention was given to prevent bench contamination of LAMP product between runs.

### Ethics statement

All patient tissue samples were acquired from the IRB approved Koschmann Brain Tumor Tissue Repository at the University of Michigan (REP00000067). Informed consent was obtained from each patient for University of Michigan research autopsy and samples were deidentified before being added to the repository.

### Reporting summary

Further information on research design is available in the Nature Research Reporting Summary linked to this article.

## Supplementary information

Supplementary Information

Description of Additional Supplementary Files

Supplementary Data 1

Reporting Summary

## Data Availability

All sequencing data (fastq) generated for this work has been uploaded to the NCBI SRA database under accession PRJNA728983. Source data underlying main figures are presented in Supplementary Data 1, and uncropped versions of gels are presented in Supplementary Figure S10. Fast5 files and other data are available upon reasonable request.

## Code availability

All scripts and code used in this evaluation are available in the following GitHub repository: https://www.github.com/jackwadden/UltraRapidSeq (10.5281/zenodo.6657900). The LAMPrey tool is available in the following GitHub repository: https://www.github.com/jackwadden/lamprey (10.5281/zenodo.6657885). Guppy version 4.2.2 and LAMPrey commit 3fe89f was used to generate data for Figs. 4e, f, g, h and 5a, b, c. Guppy version 5.0.11 and LAMPrey commit 3537ac5 was used to generate data for Fig. 5e. Minimap2 version 2.17 and samtools version 1.7 were used for all relevant analyses.
