# Supplementary material for: Ultra-rapid somatic variant detection via real-time targeted amplicon sequencing
Source: Commun Biol. 2022 Jul 15;5:708. doi: 10.1038/s42003-022-03657-6 (PMC9284968; doi:10.1038/s42003-022-03657-6)
Supplement: Supplementary file 1 — Supplementary Information [file 42003_2022_3657_MOESM1_ESM.pdf]

Supplementary Material for Ultra-Rapid Somatic Variant Detection via Real-Time Targeted Amplicon Sequencing

Survey of Prior Ultra-Rapid Molecular Diagnostics

Technique	Detection Method	Detectable Cancer Markers	Reportable Target Mutations	Fastest End-to-end Result	Targeted Oligonucleotide Amplification	Quantitative Variant Fraction (*inferred)	Streaming Output
IHC	Stain	Tumor Associated Antigen	0	20-30 mins[1,2]	n	n	n
FISH	Stain	Allele specific mutation, CNVs	-	65 min[3]	n	n	n
Raman Spectroscopy	Spectroscopy	*IDH* Mutant or not	-	15 min[4]	n	n	n
RT-qPCR	Fluorescence	Gene Expression	-	32-42 min[5,6]	y	n	y
qPCR	Fluorescence	Allele Specific Point Mutation, CNVs	2-7	60-65 min[7,8]	y	y*	y
Sanger	Sequencing	Point mutations, CNVs	**4^n	4.5hr[7]	y	y	n
Illumina	Sequencing	Point mutations, CNVs	**4^n	17 hrs[9]	y	y	n
PacBio SMRT	Sequencing	Point mutations, CNVs	**4^n	>4hrs	y	y	y
ONT MinION Ligation	Sequencing	Point mutations, CNVs	**4^n	2.5hrs[10]	y	y	y
ONT MinION Rapid	Sequencing	Low-coverage WGS methylation fingerprint	-	1.5-2.6hrs[11]	n	n	y
**This Work**	**Sequencing**	Point mutations, **CNVs	****4^n**	**<30 min**	**y**	**y**	**y**

Table S1. **Molecular diagnostic techniques and associated rapid time-frames**. Sequencing-based diagnostics have not been accomplished within the intra-operative time-frame. *qPCR variant allele fractions are inferred from comparison to wildtype allele amplification or a standard curve and have not been demonstrated intra-operatively. **Sequencing can identify any mutation that lies within an amplicon; therefore, the number of reportable target mutations is at least 4^*n* (where '*n*' is the length of the amplicon(s) under evaluation*)*. ***This work does not evaluate copy number variation detection, but should be capable of detecting these types of mutations given the addition of an assay targeting a copy-number control gene. FISH = Fluorescent In-Situ Hybridization; IHC = Immunohisto Chemistry; RT-qPCR = reverse transcription quantitative polymerase chain reaction; qPCR = quantitative polymerase chain reaction.

Supplementary Note 1: PCR and LAMP Primer Sets, Melting Temperatures, and Cycling Parameters

Primer sets used in all evaluations are listed in Table S1 below. ONT handshake sequence tails are highlighted in red and blue. For tailed primers, both the genomic length and total amplicon

length including the handshake sequences (bolded) are listed. For FIP and BIP primers, poly-T bend segments are bolded.

Primer Set	Genome Coverage	Amplicon Size	Name	Sequence
3-step 146	146	190	Fwd	**TTTCTGTTGGTGCTGATATTGC**CTGCAGGCAAGCTTTTCT
			Rev	**ACTTGCCTGTCGCTCTATCTTC**AGGCTTTTTCACGCCG
3-step 216	216	260	Fwd	**TTTCTGTTGGTGCTGATATTGC**GAATCAGCAACTCGGTCGAC
			Rev	**ACTTGCCTGTCGCTCTATCTTC**CAGGCAAGCTTTTCTGTGGT
3-step 565	565	609	Fwd	**TTTCTGTTGGTGCTGATATTGC**AGCTTCCGAATCAGCAACTC
			Rev	**ACTTGCCTGTCGCTCTATCTTC**TTCCCTAATCGTCTCCAATATTCTC
3-step 910	866	910	Fwd	**TTTCTGTTGGTGCTGATATTGC**CTCCTGCAGCGCCATCA
			Rev	**ACTTGCCTGTCGCTCTATCTTC**AGTCTCCCTCTCGCCCAA
2-step	254	298	Fwd	**TTTCTGTTGGTGCTGATATTGC**AGGCTTTTTCACGCCGCCGGTA
			Rev	**ACTTGCCTGTCGCTCTATCTTC**GGACCAATCCAAGAAGGGCGCGGGG
Pig TP53			Fwd	**TTTCTGTTGGTGCTGATATTGC**TCTGTTCGCTCTCCATCCTC
			Rev	**ACTTGCCTGTCGCTCTATCTTC**CCACCTCGGTCATGTACTCT
HIST1H3B LAMP			F3	GATCGGTCTTGAAGTCTTGG
			B3	ATAGTTGGTGGTCTGACTCTAT
			FIP	AAAGCCTCACCGTTACCG**TTTT**CCGAATCAGCAACTCG
			BIP	GAGCAGCCTTGGTAGCCAG**TTTT**GGCTCGTACTAAACAGACAG
			FLP	GAGATCCGCCGCTACCAA
			BLP	TTACCGCCGGTGGATTTC
H3F3A LAMP			F3	GTTTGGTAGTTGCATATGGTG
			B3	ATACCTGTAACGATGAGGTTTC
			FIP	GCGGGCAGTCTGCTTTGTA**TTTT**ATGCTGGTAGGTAAGTAAGGA
			BIP	CGACCGGTGGTAAAGCACC**TTTT**CACCCCTCCAGTAGAG
			FLP	CGAGCCATGGTACAGAGAC
			BLP	CAGGAAGCAACTGGCTACA

Table S2. **All primer sequences used in experiments in the paper and supplementary materials**.

Experiment	Initial Denature	Cycles	Denature	Anneal	Extend	Final Extend
3-step 146 length eval	30s @ 98°°C	35	10s @ 98°C	15s @ 67.8°C	40s @ 72°C	120s @ 72°C
3-step 216 length eval	30s @ 98°C	35	10s @ 98°C	15s @ 64°C	40s @ 72°C	120s @ 72°C
3-step 565 length eval	30s @ 98°C	35	10s @ 98°C	15s @ 65°C	40s @ 72°C	120s @ 72°C
3-step 866 length eval	30s @ 98°C	35	10s @ 98°C	15s @ 70.7°C	40s @ 72°C	120s @ 72°C
2-step eval	30s @ 98°C	28	5s @ 98°C	10s @ 72°C	-	30s @ 72°C
3-step 216 end-to-end	30s @ 98°C	28	5s @ 98°C	5s @ 64°C	8s @ 72°C	30s @ 72°C
4PP 3-step 216 eval	30s @ 98°C	35	5s @ 98°C	10s @ 64°C	13 @ 72°C	30s @ 72°C
LAMP end-to-end	-	-	14m @ 65°C	-	-	-

Table S3. **Cycling parameters used for all experiments.**

Supplementary Note 2: Evaluation of Impacts of Amplicon Size on Rapid Library Preparation Performance

To evaluate the feasibility of using the rapid library preparation kit on short amplicons we designed, amplified, and sequenced various lengths of tailed PCR amplicons (187bp,

260bp, 609bp, 910bp) using the rapid library kit. Primers were designed to cover the *HIST1H3B K27M* hotspot mutation present in pediatric brain tumor samples available to us via a biorepository (see Methods section). *HIST1H3B K27M* and *H3F3A K27M* are recurrent hotspot mutations in pediatric high-grade gliomas and predict especially poor prognosis.

We were successfully able to sequence and align all amplicons using standard bioinformatics pipelines (see Methods section). However, various inefficiencies were noted (Figure S1a). Some sequenced reads were not able to be successfully mapped to the human genome. This loss was between 8%-20% (Mapping Loss; Figure S2b) among all amplicon sizes and decreased as the amplicon length increased. This indicates that longer amplicons tend to fragment into longer reads which are generally easier to map.

Based on the final amplified mass relative to the input template genomic DNA (measured via Qubit, Invitrogen dsDNA HS Assay #Q33230), we also would expect amplicons to be sequenced in roughly the same proportion. However, we observed a bias away from this expectation in favor of background genomic DNA (Expectation Letdown; Figure S1b). This indicates the transposome favors interacting with, and fragmenting longer genomic reads versus shorter amplicons[12]. While a trend favoring longer amplicons exists, even the shortest amplicon (187bp) incurred a loss of only 6.9% (versus 2.2% for the 910bp product) relative to amplified mass, an acceptable loss.

Fragmentation loss (Fragmentation Loss; Figure S1b)—defined here as the reads that map to the target region but do not cover the target locus—is notably worse for longer amplicons. This is due to longer amplicons fragmenting in multiple locations, producing a higher ratio of strands in the library that do not contain the target locus. The transposome will also fragment amplicons near their ends, producing both nearly full-length amplicons, and fragments that are too short to map properly map. This leads to an uneven distribution of aligned read lengths (Figure S1c)—especially for shorter amplicons—with informatics favoring longer strands >150bp producing higher target coverage near the middle of amplicons (Figure S1d/e).

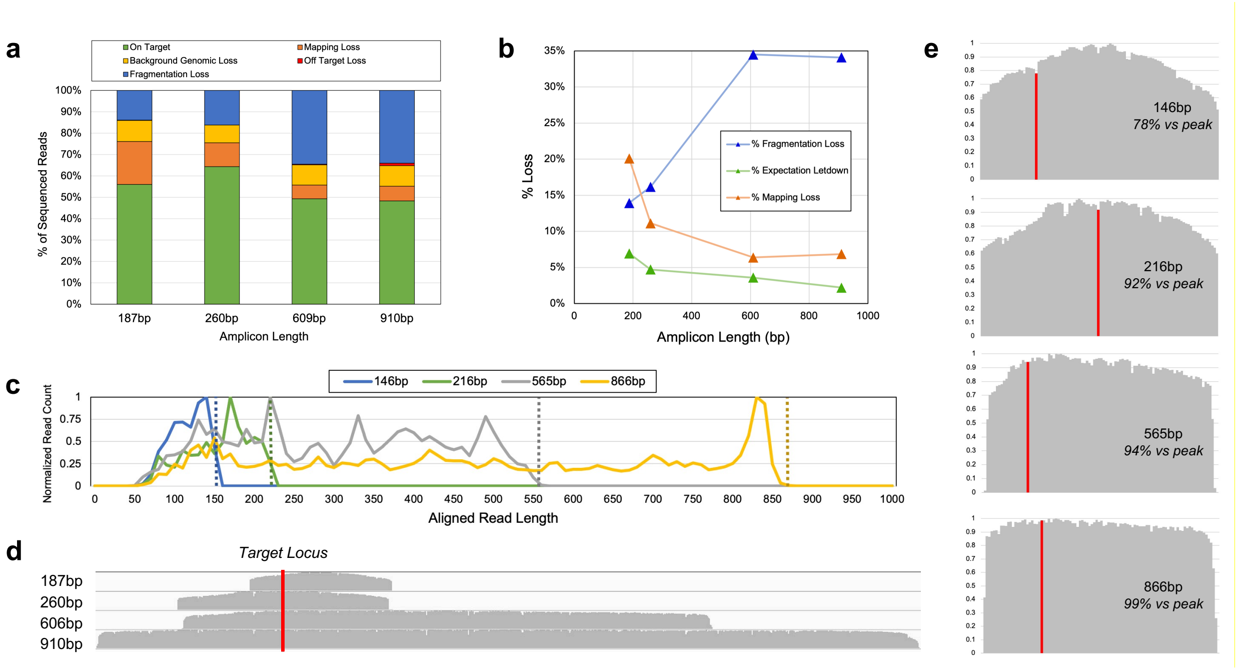

Figure S1: **Fragmentation-based library preparation and its behavior when sequencing short amplicon products**. **(a)** Due to the presence of background genomic DNA, fragmentation-based library preparation, and difficulty mapping short amplicon fragments, many sequenced reads are not diagnostically relevant, leading to additional sequencing time required to reach a desired target depth. **(b)** Longer amplicons tend to fragment into longer reads, which are easier to map (Mapping Loss). Longer amplicons and genomic DNA also tend to be favored by the transposome when compared to shorter amplicons (Expectation Loss). However, longer amplicons can fragment multiple times, producing a higher proportion of read fragments that do not contain a particular target in the library (Fragmentation Loss). **(c)** Aligned read lengths for all amplicons. Shorter amplicons lead to a larger proportion of reads that are too short to align properly. **(d)** Aligned read coverage histograms for all amplicons. **(e)** Width normalized coverage histograms for each amplicon showing edge penalties when the location of interest is near the end of amplicons. These results indicate it is beneficial to design primer sets that generate relatively short amplicons with any hot-spot target of interest near the center of the amplicon (to reduce fragmentation loss), but long enough to preserve the proportion of useful, mappable reads.

Supplementary Note 3: PCR Parameter Optimization and Efficiency Evaluation

Faster PCR cycle parameters can lead to significant reductions in end-to-end amplification time. Using the best performing primer set evaluated (260bp) we performed cycle parameter optimization, reducing cycle times[13] while balancing reaction efficiency. Due to the relatively small amplicon size allowed by the ONT rapid library preparation chemistry, PCR cycle times were able to be significantly reduced versus prior work[10] but did end up relatively close to suggested lower-bound times (Figure S2a). Estimated end-to-end PCR times were modeled based on 3C/s ramp rate (Figure S2b). PCR paper time ignores ramp rate penalty and any handling penalties.

Optimized PCR time is dominated by ramp rate of standard lab thermocyclers, and could be further reduced by using more rapid thermocyclers.

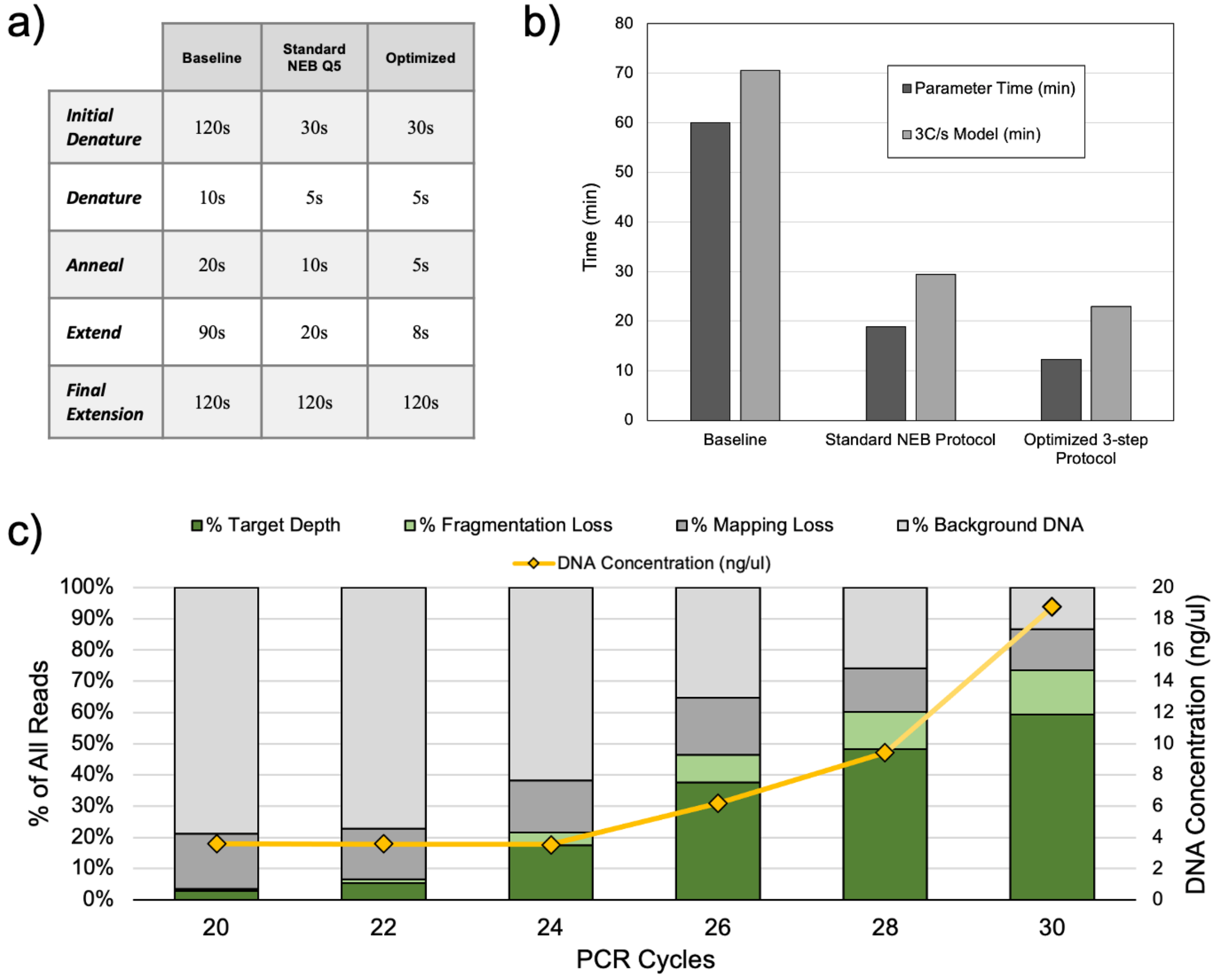

	Baseline	Standard NEB Q5	Optimized
Initial Denature	120s	30s	30s
Denature	10s	5s	5s
Anneal	20s	10s	5s
Extend	90s	20s	8s
Final Extension	120s	120s	120s

Figure S2. **3-step PCR assay optimized parameters, comparison to baseline protocols, and performance over various cycle counts. (a)** cycle times for a baseline amplification protocol[10], the standard NEB protocol, and our our optimized parameters. **(b)** on-paper times differ significantly from modeled and measured times during experimentation. As cycle parameters are reduced, thermocycler ramp time begins to dominate total amplification time leading to a >50% efficiency loss (~10min penalty) and a reduction in the marginal benefit of cycle time reduction. **(c)** PCR performed well, producing a high-proportion of on-target amplicons after 26 cycles.

The proper cycle threshold for optimal end-to-end diagnostic performance can be estimated by sequencing product from PCR with varying numbers of cycles. We performed PCR using 1:10 diluted LQE extracted DNA (see Methods section). We then measured both the resulting PCR product concentration via Qubit fluorometer (Invitrogen; HS dsDNA Assay; #Q33230) and sequenced each resulting product using a barcoded, multiplexed rapid library preparation methodology (ONT; SQK-RBK004). Resulting reads were aligned to the human

reference and classified according to alignment (see Methods section). Over time, we see both the total mass, as well as the proportion of PCR product relative to background genomic reads grow (Figure S2c).

Supplementary Note 4: Impact of Rapid Adapter Incubation Time

We tested four different rapid adapter incubation times from 5 minutes to 2 minutes. Five minutes is the incubation time recommended by ONT's Rapid Barcoding library preparation kit (SQK-RBK004). Reactions were started at successive 1-minute intervals to assure all incubations ended at the same time. Once incubations finished, each reaction was immediately and independently mixed with ¼ ONT pre-mixed sequencing mix. This was to prevent ONT loading beads from preferentially binding to amplicons first added to one pooled sequencing mix, as observed in earlier experiments. All reactions were then mixed, combined, and sequenced until the lowest target depth reached >5,000 target reads. Fast5 files were basecalled using Guppy V4.2.2 and aligned using Minimap[14] v2.17. Adapter efficiency was estimated by comparing the relative read counts of various time points. No upward trend in adapter efficiency was noted (Figure S2), indicating that a 2-minute incubation time is sufficient to create a library of sufficient quality to sequence and rapidly diagnose hotspot mutations.

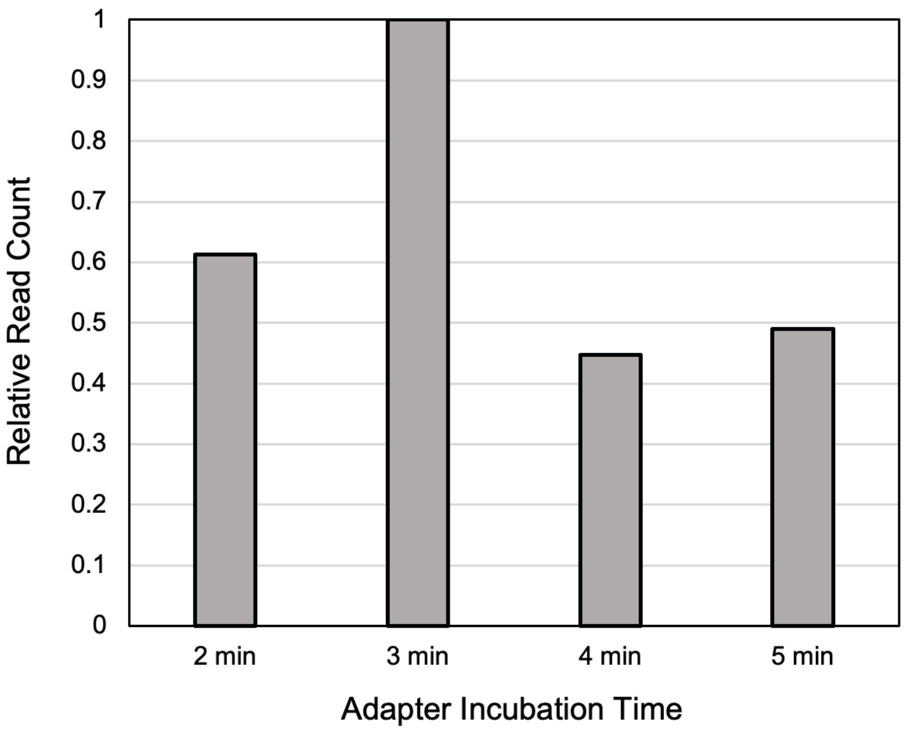

Figure S3. **Relative read counts from four different Rapid Adapter (RAP) incubation times.**

Supplementary Note 5: LQE Dilution Efficiency and MinION Sequencing Feasibility

To test the impacts on dilutions of the Lucigen QuickExtract (LQE) extracted DNA on PCR efficiency, two primer sets were designed to target exon 5 of the porcine (sus scrofa) TP53 gene. We used representative aliquots (~20mg) of pig brain tissue and followed the standard extraction protocol. Serial dilutions of extract were made and amplified using the following cycling parameters: 30s @ 98°C; then 35 cycles of 10s @ 98°C, 10s @ 62°C, 20s @ 72°C; then a final extension of 2 minutes @ 72°C. Amplification efficiency was measured via inspection of band brightness following gel electrophoresis (Figure S3). Dilutions of 8x-32x were substantially more efficient than 1x-4x with a 10x dilution (suggested by the manufacturer) performing the best across both primer sets.

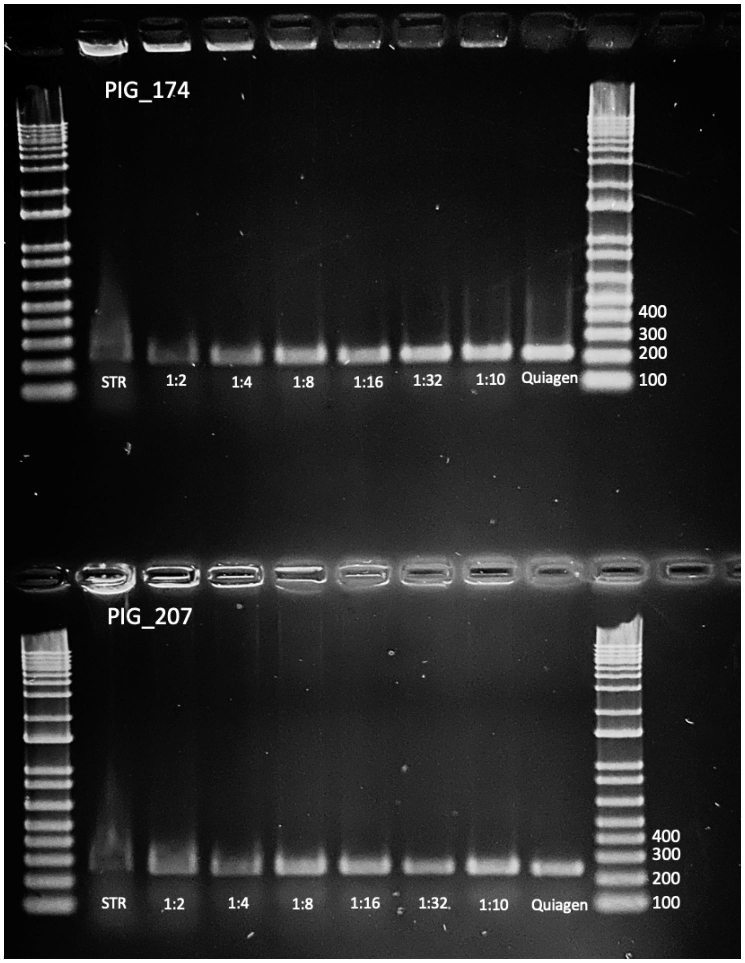

Figure S4. **Gel electrophoresis of PCR product from various dilutions of DNA extracted from Lucigen QuickExtract DNA extraction solution.** 10x (1:10) dilution performed best across both trials and was selected as a standard dilution for all further experiments.

We then performed an extraction on an aliquot of pediatric DIPG tumor and normal brain tissue following the standard LQE protocol and performed PCR with 10ul of 10x diluted DNA (1ul LQE product in 9ul nuclease free water). After confirming amplification, we sequenced product from 26 cycles of PCR to confirm that this product was sequence-able and produced valid reads and identified the known *HIST1H3B K27M* variant. We were able to successfully sequence and confirm the variant using our standard informatics pipelines, basecalling using ONTs Guppy basecaller and aligning reads to the human reference using minimap2 version 2.17. A read pileup visualized using integrated genome viewer is shown below.

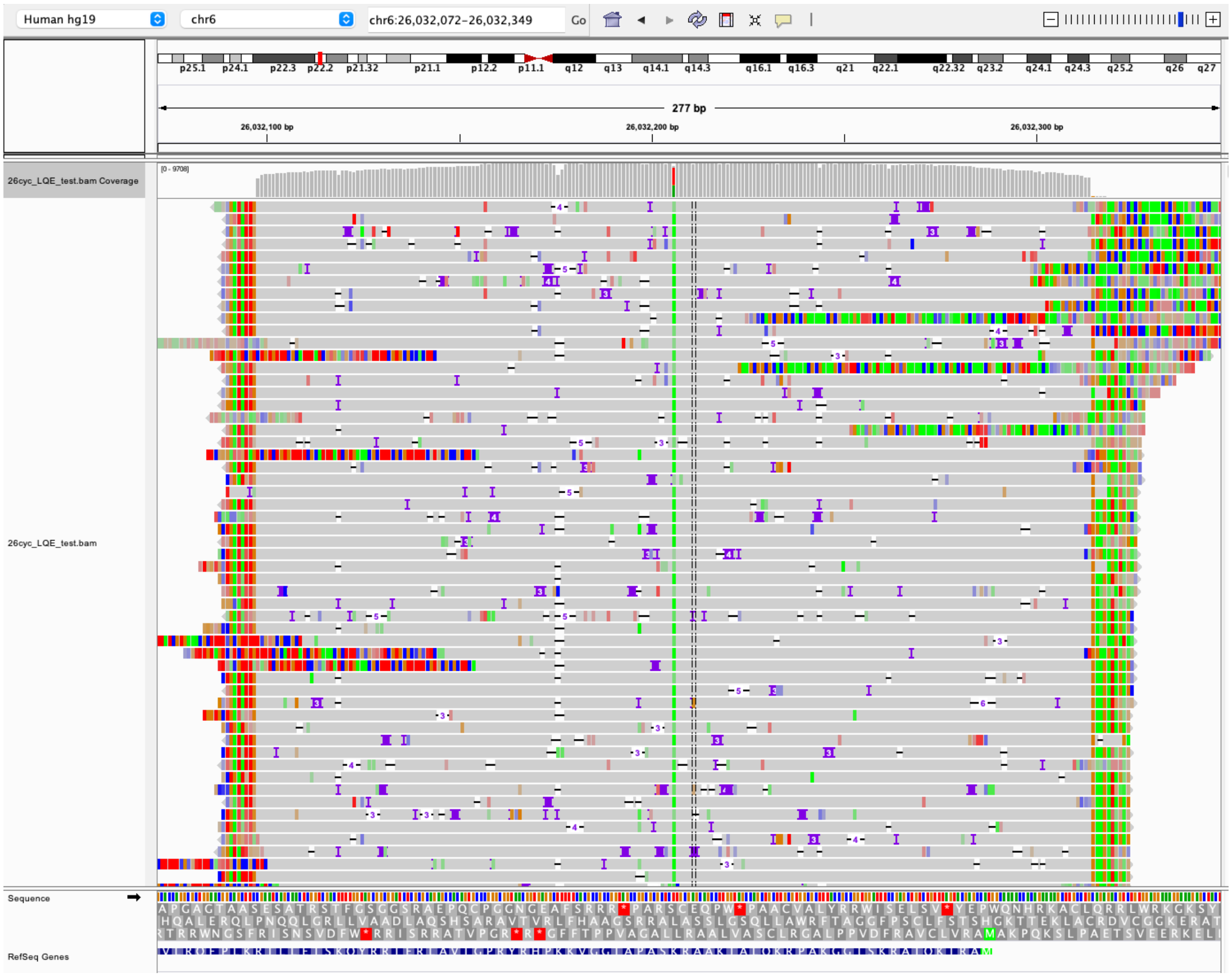

Figure S5. **Visualization of read pileup generated by Integrated Genome Viewer (IGV 2.8.0) showing fragmented amplicons aligned to *HIST1H3B.* The somatic *K27M* mutation is visible near the center of the alignment.**

Supplementary Note 6: LQE Incubation Time Efficiency

DNA from prior standard protocol extractions was compared to DNA extracted from a separate aliquot of tumor tissue from the same patient. Extracted DNA was amplified using our primary *H3F3A K27M* LAMP assay in a Eppendorf Mastercycler, prepared using the suggested amount

of dye. Amplification and fluorescence monitoring was performed at 65°C on an Eppendorf Mastercycler with 15s measurement intervals (cycles). Fluorescence curves are shown in Figure S4. The assay showed almost identical performance between the two extraction methodologies. Note that due to the inability to pre-heat the real-time fluorescence machine, and the difficulty of identifying the ratio of background DNA to the amplifying target, time-to-amplification and the sequencing threshold seems to be underestimated compared to our ultra-rapid protocol determined by time-sweep sequencing.

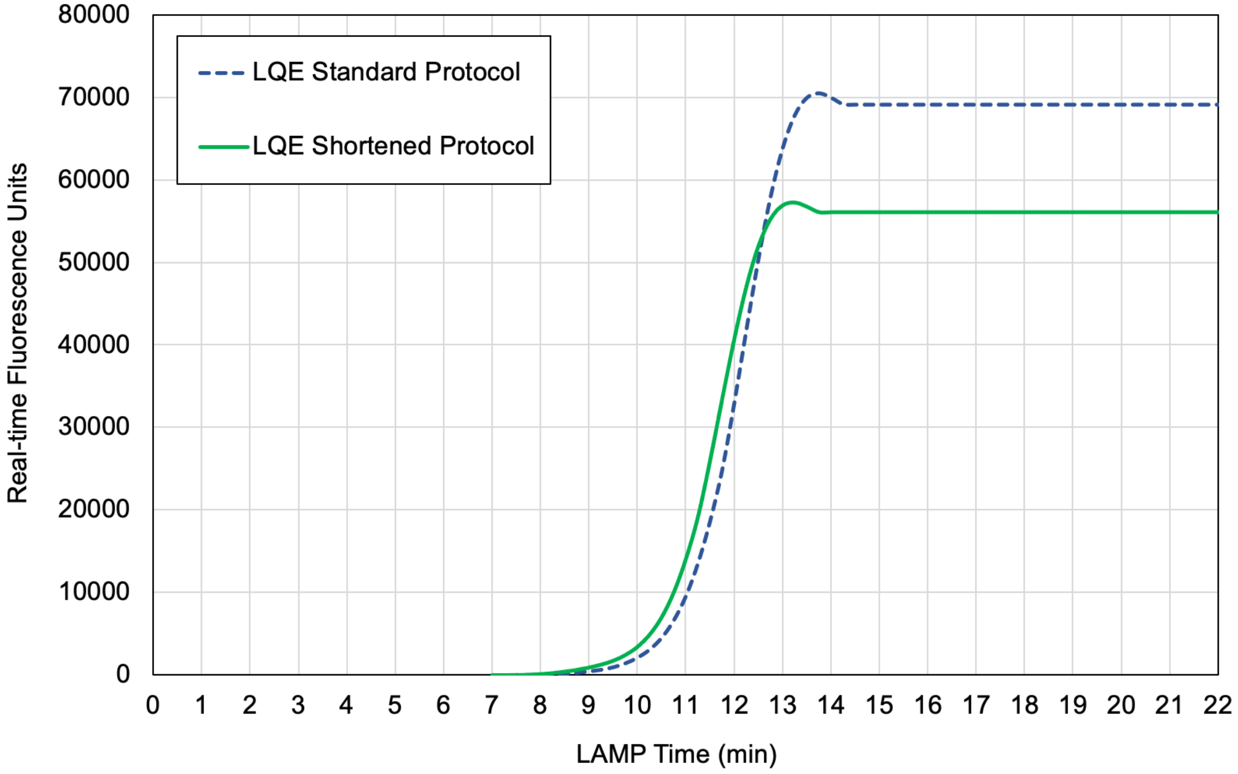

Figure S6. **Real-time amplification curves of DNA extracted using the standard LQE protocol and the shortened version.** The standard protocol leverages a 6 minute incubation time and the shortened protocol leverages a 1 minute incubation time. Amplification between the two methods is almost identical indicating that a reduced extraction time does not impact amplification rate, either due to reduced inhibitor concentration or unnecessarily long incubation time for this tissue type.

Supplementary Note 7: Diagnostic Time Optimization Approach

Target amplification increases the target signal over background genomic noise and typically involves conservative “end-point” protocols that maximize amplification (e.g. 35+ PCR cycles) at the expense of time. For a time-optimal diagnostic, amplification should only be performed if the time-to-result benefit outweighs the time cost of further amplification. We used an analytical model

to identify this “worthwhile” threshold for amplification time and suggest protocols that attempt to optimize for time-to-result.

As an illustrative example of our method, consider perfectly efficient PCR-based amplification where the target amplicon mass doubles every cycle. Given any amount of input DNA from any number of PCR cycles, we can model total diagnostic time using the formulas shown in Figure S7a. As amplicon mass grows, the proportion of total mass that is background genomic material (gDNA) reduces (Figure S7b) and thus the sequencing rate of target amplicons (versus background genomic DNA) increases. Assuming a 250x target coverage requirement for diagnosis, at a certain point, it becomes a waste of time to continue amplification, and instead becomes worthwhile to begin sequencing. Figure S9c shows an illustration of this trade-off using optimistic sequencing and PCR cycling parameters and a more sophisticated model of sequencing time (Table S4). Assuming perfect PCR efficiency, the optimal amount of amplification is 16 cycles before further amplification results in wasted diagnostic time. This represents a savings of ~12-22 minutes over a typical PCR-protocol.

To apply this methodology in practice, we first estimate useful target amplicon fraction and ONT flow-cell sequencing rates experimentally for a particular assay and tissue type (e.g. Figure S9a), and then use the sequencing performance model to suggest amplification times that aim to produce a time-optimal result (Figure S7b).

a

$$T_{total} = T_{amp} + T_{seq} \quad (1)$$

$$T_{amp} = T_{init} + T_{cycle} \times N_{cycle} + T_{final} \quad (2)$$

$$F_{target} = \frac{2^{N_{cycle}}}{2^{N_{cycle}} + N_{background}} \quad (3)$$

$$T_{seq} = N_{depth} \times \frac{1}{N_{pores} \times R_{sample} \times F_{target}} \quad (4)$$

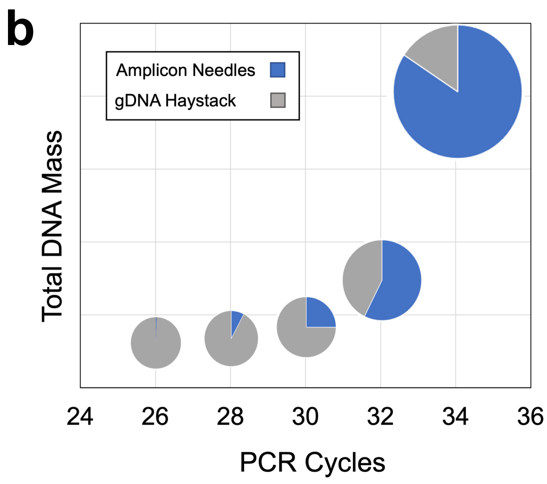

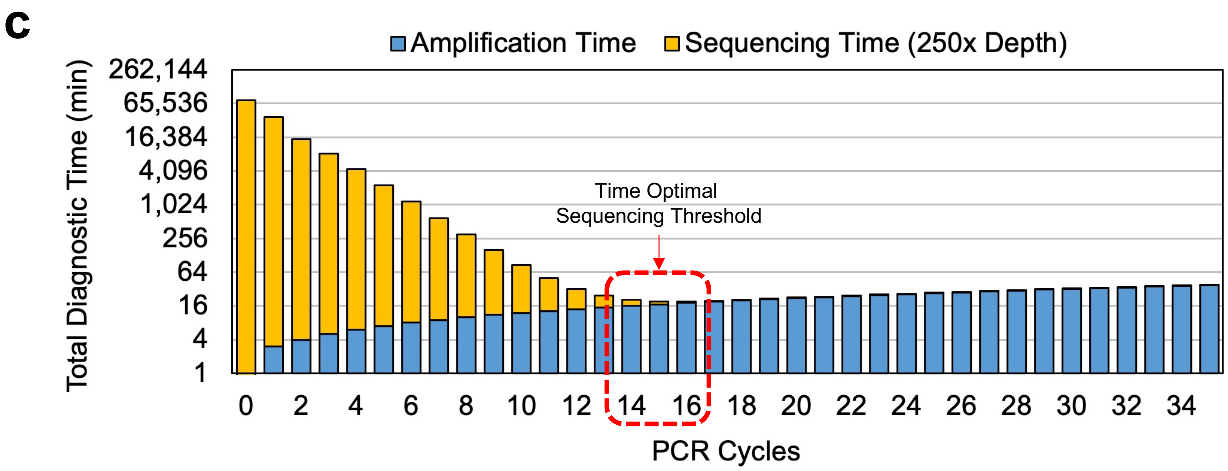

Figure S7. **Amplification characterization and sequencer performance modeling hels identify the amount of amplification time or cycles required to minimize total diagnostic time. (a)** Diagnostic time can be modeled as the sum of amplification time (T_{amp}) time required to sequence to a target diagnostic depth. Amplification time (T_{amp}) is a function of cycling parameters in the case of PCR or incubation time in the case of LAMP. Sequencing time is a function of the target depth requirement (N_{depth}), available parallel sequencing pores (N_{pores}), the read sampling rate per pore (R_{sample}), and the fraction of all sampled reads that are diagnostically useful (F_{target}). **(b)** Over time, both total mass and proportion of amplicons relative to background genomic template (F_{target}) increases. As amplicon "needles" increase, less sequencing time is required to search for them in the background genomic DNA (gDNA) "haystack." **(c)** Using example PCR parameters, we can model total diagnostic time, and identify an amplification threshold that results in a minimal time-to-result.

Optimal Amplification Threshold Sequencing Model Example Parameters

Figure S9 develops a model framework to analytically derive the optimal amount of time spent on amplification that results in a minimal total diagnostic time (the optimal amplification threshold). The equations used to build the model and the corresponding input parameters used to generate Figure S9 are shown below in Table S4.

Parameter	Description	Value or Equation
N_{depth}	Diagnostic coverage requirement	250x
T_{cycle}	PCR Cycle Time	60s
T_{init}	PCR initial denaturation time	60s
T_{final}	PCR final extension time	60s
N_{pores}	Active Channels/Pores Contributing to Sampling	512
R_{seq}	Transposition rate of DNA through the Nanopore	400bp/s
$T_{capture}$	Average Capture Time (time-between read capture per pore)	2s
$L_{background}$	Average Background Read Length	4kbp
L_{target}	Target amplicon length	250bp
$L_{background}$	Background Genomic Size	3,079,843,747bp
$N_{background}$	The number of background reads	$N_{background} = \frac{S_{background}}{S_{read}}$
N_{target}	The number of targets	$N_{target} = 2^{cycles}$
F_{target}	The fraction of target amplicons relative to background genomic reads	$F_{target} = \frac{N_{target}}{N_{background} + N_{target}}$
L_{read}	Average sequenced read length	$L_{read} = F_{target} * L_{target} + (1 - F_{target}) * L_{background}$
R_{sample}	Average sampling rate (reads/pore/second)	$R_{sample} = 1/(\frac{L_{read}}{R_{seq}} + T_{capture})$
T_{seq}	Time to sequence to target depth	$T_{seq} = N_{depth} * (\frac{1}{N_{pores} * R_{sample} * F_{target}})$
T_{amp}	Amplification time (PCR)	$T_{amp} = T_{init} + T_{cycle} * N_{cycle} + T_{final}$
T_{total}	Total diagnostic time (amplification + sequencing)	$T_{total} = T_{amp} + T_{seq}$

Table S4. **Model parameters and equations used to generate Figure S9C.**

Supplementary Note 8: Statistical Methods for Statistical Significance of Variant Call

For our timed evaluations of the ultra-rapid sequencing protocols, we ended the protocol once read support over the target locus (mutant + wildtype calls) reached 250x. This is an arbitrary threshold and might not be enough coverage to call some very low variant allele fractions. In practice, *H3 K27M* fractions from tumor samples are high (20-60%). In this case, 250x is more than enough to call the variant with 99.99% confidence.

Statistical significance of a variant can be identified in real time, allowing Optimal Amplification Threshold Sequencing to be applied without an arbitrary coverage requirement. Our methodology involves computing the confidence interval for a proportion for each alternate variant call, or a targeted variant in the case of our *H3K27M* assay. The formula for computing the confidence interval is shown below as Equation 5:

$$\hat{p} \pm z \cdot \sqrt{\frac{\hat{p}(1-\hat{p})}{n}} \qquad \textbf{\textit{(5)}}$$

Where $\hat{p}$ is the observed variant proportion, n is the target support (mutant and wild-type calls), and z is the Z-value for the desired confidence level.

Given that ONT sequencers and corresponding basecallers have relatively high error rates, this confidence interval can be compared to the confidence interval for the expected error rate for that context. We call a variant statistically significant when the lower-bound confidence interval for the observed variant proportion exceeds the upper-bound confidence interval for the expected error rate proportion. The error rate for this locus is known to be ~1% based on prior characterization in Bruzek et al.[15], but we conservatively assume 1.5% for this analysis. Sequencing and basecaller errors are highly dependent on oligonucleotide sequence context and are much higher in low-complexity regions featuring homopolymers. This context-dependent error rate should be considered when calling variants in such regions. An example for a VAF proportion of 10% is shown in Figure S5. Alternatively, a healthy, wildtype sample can be pre-sequenced to characterize the error rate for a particular tissue, assay, target locus, and basecaller version. Future basecalling accuracy improvements will help reduce this error and lower sequencing depth requirements.

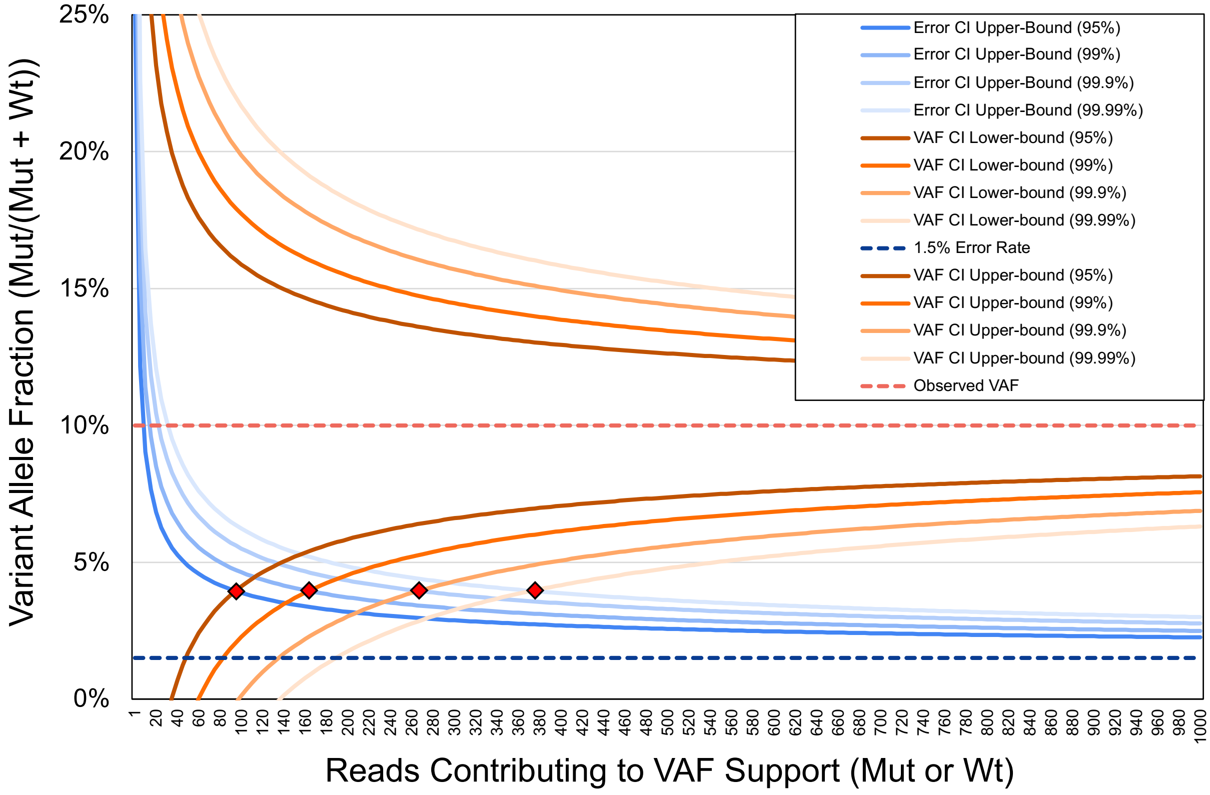

Figure S8. **Confidence intervals for simulated observed VAF of 10% (orange) relative to the confidence interval for estimated background 1.5% error rate (blue) plotted for various desired confidence levels (95%, 99%, 99.9%, 99.99%).** For each confidence level, we identify a variant call as statistically significant when the lower bound confidence interval of the observed variant fraction becomes larger than the upper bound confidence interval of the background error (red diamonds). Underlying VAF, error rate for that context, and desired confidence level greatly impact the locus support required to call a variant. In practice, it is best to apply these tests in real time. Even for a relatively low VAF of 10%, ~270 reads is expected to be sufficient to call the variant with 99.9% confidence.

Supplementary Note 9: End-to-End PCR-based Ultra-rapid Protocol

The final end-to-end protocol is available in updateable electronic format at protocols.io (https://www.protocols.io/view/ultra-rapid-sequencing-pcr-bs7bnhin). A visual representation approximating the timeframe of the protocol as performed in this work is shown in Figure S7.

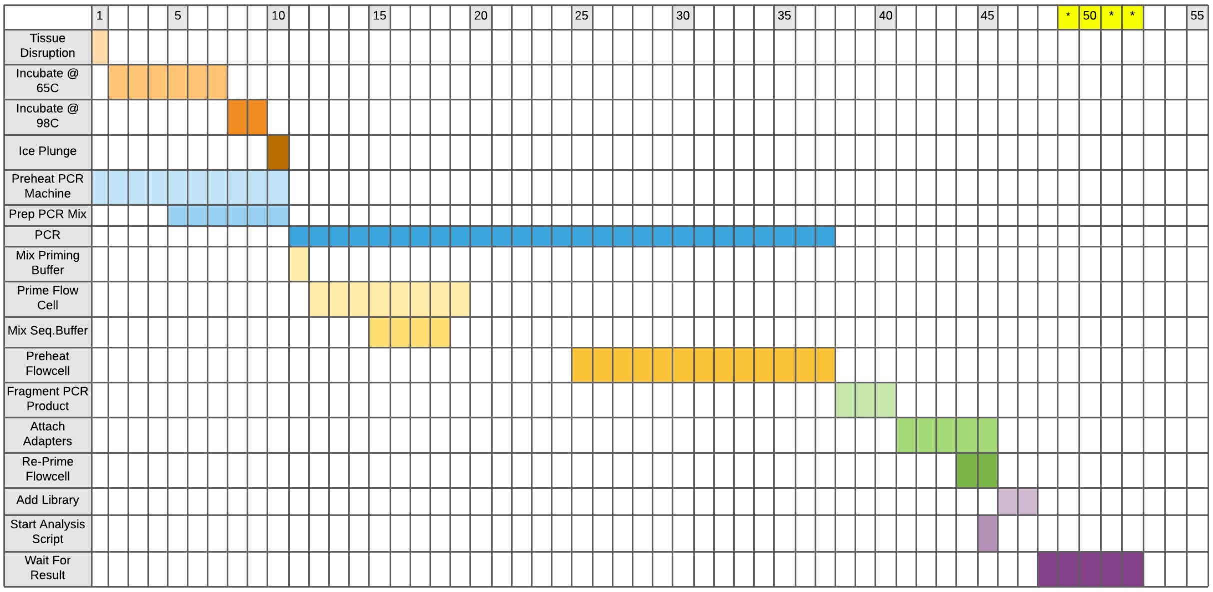

Figure S9. **Approximate protocol timeline showing timeframes and overlap of certain steps.**

Unedited H3F3A and HIST1H3B LAMP Product Visualized via Gel Electrophoresis

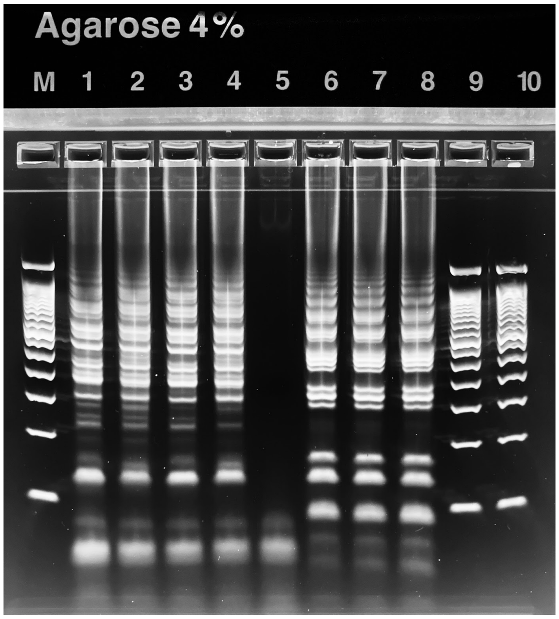

Figure S10. **Gel electrophoresis of LAMP product using two different assays.** Lanes M, 9, and 10 are E-gel 50bp ladders (Invitrogen #10488099). Lanes 6-8 are H3F3A targeting LAMP product. Lanes 2-4 are HIST1H3B targeting product. Lane 9, 2-4, and 6-9 constitute Figure 3b and 3c in the main paper.

Supplementary Note 10: LAMPrey Algorithm Description

LAMPrey is an algorithm for processing a set of FASTQ reads, attempting to identify properly formed LAMP concatemers (lamplicons) and call variants based off of a pileup of lamplicon sub-reads. As input, the user supplies a FASTQ file of sequenced LAMP product and a corresponding primer sequence file that details the 6-8 design sequences identified in LAMP primer design (B3, B2, B1, F1, F2, F3, BLP, FLP), as well as other sequences of interest such as ONT barcodes or adapter sequences. The user also describes a "target" sequence that spans the location where the mutation of interest lies. The user also specifies where this target sequence lies in the LAMP design sequence schema. A target sequence can exist anywhere between the F2 and B2 primers as long as it is not covered by another primer.

Sequence Alignment: LAMPrey first parses the FASTQ read file and optionally extracts the reads according to the ONT Guppy basecaller emitted *start_time* timestamp. This is assumed to be the approximate order and time that the reads are generated. LAMPrey then considers each read and aligns each sequence in the primer file to the read using a standard Smith Waterman alignment. Alignments are recorded if their identity is above a certain threshold (default of 75%). Once all possible primers are marked, overlapping aligned sequences are removed, prioritizing alignments with higher identity. After sequence alignment, reads can be immediately classified into one of five different categories.

Classification: If the read does not contain any aligned target sequences, it is diagnosed as either an amplicon fragment, background genomic DNA, spurious LAMP amplification, an ONT fragment leftover from library preparation, or Unknown (further broken down into "short" unknown sequences (<60bp) and other Unknown). Amplicon fragments are those that align to the gene or target region of interest within some expected distance (e.g. 1000bp) but do not contain the target sequence. These reads are expected given the fragmentation step included in library preparation. If the read aligns somewhere else in the human genome, it is marked as a background genomic read. These are also expected given that our protocol optimization approach attempts to skip target enrichment to save diagnostic time. If a read has a large proportion of primer sequences covering the read, but does not contain a target sequence, it is marked as suspected spurious amplification. These are assumed to be hybridized primers or other undesired amplification that does not capture the diagnostically relevant information of the patient, but still involves the basic LAMP machinery and at least some primer sequences. These reads are not expected but are difficult to fully avoid without extensive optimization of the LAMP assay. If a read has a high

proportion of ONT related sequences covering a large proportion of the read, it is marked as an ONT fragment. ONT fragments could be unligated adapter sequences, or read fragments that contain too little target or background DNA to successfully align to the reference. If a read cannot be classified as any of the other categories, it is marked as unknown, with reads shorter than 60bp marked as “short”.

Chaining: Once a lamplicon with at least one target sequence is identified, it is further processed to divide up the possible concatemer into its sub-reads. This is accomplished by considering each target sequence and looking for expected primer sequences to the 5’ and 3’ ends based on the LAMP assay. This is analogous to the chaining of read hits in many common read mapping algorithms. For example, if a forward target sequence exists between the F1 and B1 regions, we would look to its 5’ end to find an aligned F1 sequence, and to its 3’ end to find the complement of the B1 sequence. In this way, target sequences are “extended” in the 5’ and 3’ direction until an aligned sequence that doesn’t match the expected ordering is found (e.g. an ONT barcode, adapter sequence, or FIP/BIP loop transition), or the end of the read is found. This extension defines a sub-read. Each sub read is extracted and considered separately.

Variant Calling: Once each sub-read is identified, they are separately aligned to the target gene of interest (NOTE: not the full human reference). All sequences that successfully align are converted to a pileup, and the base pairs over the target hotspot locus are considered. Because LAMP concatemers contain N target copies, and ONT sequencers and basecalling algorithms may introduce errors, the N target copies may not agree. For variant calling, we only consider lamplicons that have majority or plurality support for one call from all alignable sub-reads. For example, a lamplicon concatemer with three sub-reads aligned over the target locus with calls CCT would be considered an C call (majority and plurality). A concatemer with two target regions with calls CT would be ignored (majority) or a random basecall would be selected from the plural basecalls (plurality). Currently, variant allele fraction is computed in real time as reads are processed based off of a user supplied VCF file describing a single variant of interest, however, LAMPrey could be easily configured to identify arbitrary mutations at multiple loci.

Supplementary Note 11: Sources of diagnostic information missed by standard informatics pipeline

This investigation revealed four major failure modes of our original informatics pipeline explaining LAMPrey’s improved performance:

1. *Off-target alignment, and filtering of non-primary alignments* (Figure 6e i): sequenced lamp concatemers are sometimes mapped to off-target locations. Correct alignments can be included as secondary or supplementary alignments by the mapper but are filtered out by the standard diagnostic pipeline.
2. *Imbalanced, fragmented concatemers* (Figure 6e ii): the fragmentation-based library preparation approach can leave concatemers imbalanced, where two different-sized sub-reads exist: one shorter with the target information, and one longer without. Read mapping algorithms will typically score the longer sub-read as the primary alignment, and soft clip the shorter, diagnostically relevant section.
3. *Short, hairpin reads* (Figure 6e iii): Concatemer fragments can contain short forward/reverse segments spanning the hairpin loops of a properly formed concatemer. The read mapper struggles to find proper chains for either of these short, competing sections, leading to mapping failure.
4. *Partially spurious, malformed concatemers* (Figure 6e iii): mis-primed LAMP amplification can occur after proper concatemer extension. If fragmentation generates a read that is mostly spurious, the read mapper may fail to align the read at all, even if a small section of target diagnostic information exists in the read.

LAMPrey did miss a small percentage of targets identified by our standard pipeline (Figure 6e). Upon further inspection, these reads were missed due to LAMPrey failing to identify target seeds in the initial seeding stage due to many basecalling errors. Tuning long-read aligner parameters and investigating one of many other alignment algorithms to improve performance is one possible approach to improve recovery of diagnostically relevant information. However, the complexity of LAMP concatemers coupled with a fragmentation-based library preparation approach and the above results clearly motivates a LAMP-specific analysis and further development of tools such as LAMPrey.

Supplementary Note 12: End-to-End LAMP-based Ultra-rapid Protocol

The final end-to-end protocol is available in updateable electronic format at protocols.io (https://www.protocols.io/view/ultra-rapid-sequencing-lamp-btvmnn46). A visual representation approximating the time-frame of the protocol as performed in this work is shown in Figure S8 along with a step-by-step protocol. This final protocol combines DNA extraction, amplification, and library preparation into one thermocycler program to save time and also simplify equipment usage.

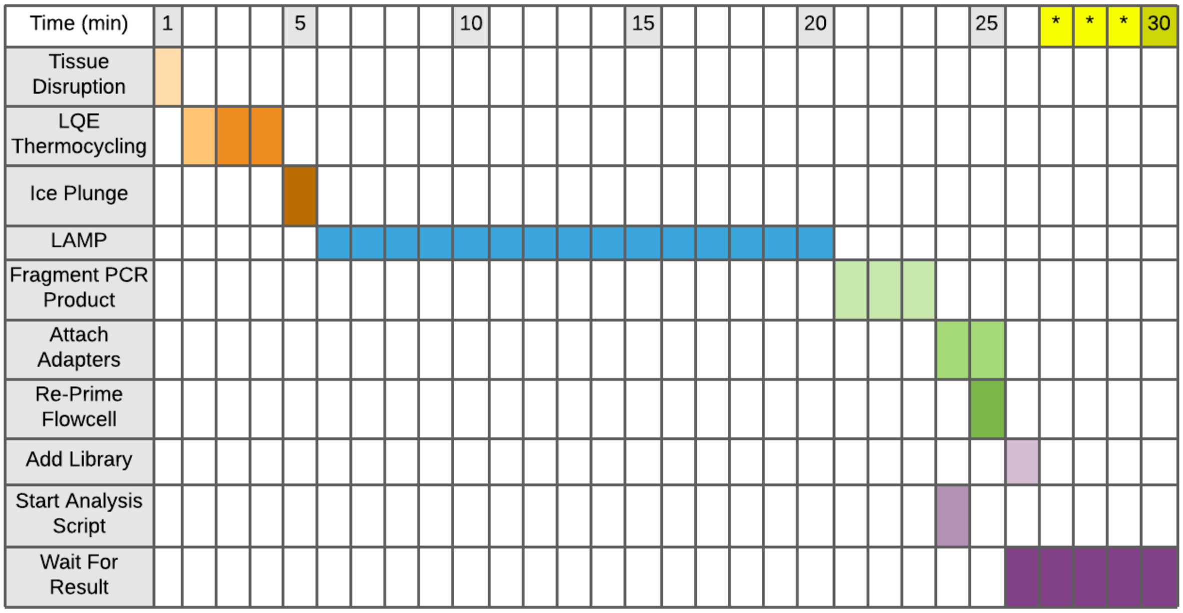

Figure S11. **Steps and time-frame of performed end-to-end LAMP-based diagnostic.** Yellow highlighted region delineates the time-frame of an expected result. We achieved a variant call at ~29.5 minutes. Flow cell priming, LAMP mix preparation, flow cell QC, sequencing buffer mixing, and fragmentation buffer preparation was all performed ahead of time.

Supplementary References

1. Terata, K. *et al.* Novel rapid-immunohistochemistry using an alternating current electric field for intraoperative diagnosis of sentinel lymph nodes in breast cancer. *Sci. Rep.* **7**, 2810 (2017).
2. Lovchik, R. D., Taylor, D. & Kaigala, G. Rapid micro-immunohistochemistry. *Microsyst. Nanoeng.* **6**, 1–10 (2020).
3. Duncan, D. J., Vandenberghe, M. E., Scott, M. L. J. & Barker, C. Fast fluorescence in situ hybridisation for the enhanced detection of MET in non-small cell lung cancer. *PLOS ONE* **14**, e0223926 (2019).

4. Livermore, L. J. *et al.* Rapid intraoperative molecular genetic classification of gliomas using Raman spectroscopy. *Neuro-Oncol. Adv.* **1**, vdz008 (2019).
5. Al-Ramadhani, S. *et al.* Metasin—An Intra-Operative RT-qPCR Assay to Detect Metastatic Breast Cancer in Sentinel Lymph Nodes. *Int. J. Mol. Sci.* **14**, 12931–12952 (2013).
6. Smith, G. J., Hodges, E., Markham, H., Zhang, S. & Cutress, R. I. Evaluation of the Metasin assay for intraoperative assessment of sentinel lymph node metastases in breast cancer. *J. Clin. Pathol.* **70**, 134–139 (2017).
7. Kanamori, M. *et al.* Rapid and sensitive intraoperative detection of mutations in the isocitrate dehydrogenase 1 and 2 genes during surgery for glioma. *J. Neurosurg.* **120**, 1288–1297 (2014).
8. Gm, S. *et al.* Rapid Intraoperative Molecular Characterization of Glioma. *JAMA Oncol.* **1**, 662–667 (2015).
9. Bamborschke, D. *et al.* Ultra-rapid emergency genomic diagnosis of Donahue syndrome in a preterm infant within 17 hours. *Am. J. Med. Genet. A.* **n/a**,.
10. Euskirchen, P. *et al.* Same-day genomic and epigenomic diagnosis of brain tumors using real-time nanopore sequencing. *Acta Neuropathol. (Berl.)* **134**, 691–703 (2017).
11. Djirackor, L. *et al.* Intraoperative DNA methylation classification of brain tumors impacts neurosurgical strategy. *Neuro-Oncol. Adv.* **3**, (2021).
12. Nextera XT Library Prep: Tips and Troubleshooting. 6.
13. Protocol for Q5® Hot Start High-Fidelity 2X Master Mix | NEB. https://www.neb.com/protocols/2012/08/30/protocol-for-q5-hot-start-high-fidelity-2x-master-mix-m0494.
14. Minimap2: pairwise alignment for nucleotide sequences | Bioinformatics | Oxford Academic. https://academic.oup.com/bioinformatics/article/34/18/3094/4994778.

15. Bruzek, A. K. *et al.* Electronic DNA Analysis of CSF Cell-free Tumor DNA to Quantify Multi-gene Molecular Response in Pediatric High-grade Glioma. *Clin. Cancer Res.* **26**, 6266–6276 (2020).
