# Supplementary material for: Ultra-rapid somatic variant detection via real-time targeted amplicon sequencing
Source: Commun Biol. 2022 Jul 15;5:708. doi: 10.1038/s42003-022-03657-6 (PMC9284968; doi:10.1038/s42003-022-03657-6)
Supplement: Supplementary file 2 — Description of Additional Supplementary Files [file 42003_2022_3657_MOESM2_ESM.pdf]

# Description of Additional Supplementary Files

**File name:** Supplementary Data 1
**Description:** Source data behind all graphs in the paper.
